# Cytochrome P450 inhibitors: friends or foes?

**DOI:** 10.3389/fchem.2026.1988344

**Published:** 2026-10-13

**Authors:** Shivam Kumar Vyas, Md Imran Ahmed, Vaibhav A. Dixit

**Affiliations:** Department of Medicinal Chemistry, Department of Pharmaceuticals, Ministry of Chemicals and Fertilisers, National Institute of Pharmaceutical Education and Research, Guwahati (NIPER Guwahati), Govt. of India, Guwahati, Assam, India

**Keywords:** CYP450, drug metabolism, inhibitor, irreversible inhibition kinetics, mechanism-based inhibition, partitioning factor

## Abstract

Cytochrome P450 (CYP450) enzymes play a central role in the metabolism of xenobiotics and endogenous compounds, influencing drug pharmacokinetics, efficacy, and safety. Inhibition of these enzymes has traditionally been viewed as a major liability in drug discovery due to its association with drug-drug interactions (DDIs), toxicity, and metabolic instability. However, inhibition of specific CYP450 isoforms has emerged as a powerful therapeutic strategy in multiple disease areas, including cancer and infectious diseases. This review presents a comprehensive analysis of data and regulatory guidance on CYP450 inhibitors to gain insights into whether CYP450 inhibition is a useful property (“a friend”) or a liability (“a foe”) for drug discovery scientists. Review of the literature shows that the design of novel CYP450 inhibitors requires an understanding of mechanisms of inhibition, whereas the clinical outcomes are mainly determined by isoform selectivity and effects of metabolism on drug action including reactive metabolite formation. The kinetic behaviour of representative inhibitor drugs is reassessed using variations in Michaels-Menten parameters *β* = *K*_m_/*K*_i_, and partitioning factor (*k*_inact_/*k*_off_). A modified classification of CYP450 inhibitors is proposed which consolidates the often-confusing classifications (mechanistic vs. spectroscopic) reported in the literature and FDA guidelines. The beneficial role of CYP450 inhibition in treatment and pharmacokinetic boosting is also discussed. Finally, advances utilising deep neural networks (DeepCYP) and machine learning methods (CYPlebrity, ML-augmented docking) in the last 5 years in inhibitor predictions are reviewed. This review provides a fresh perspective on classifying CYP450 inhibitors, existing guidelines for evaluation of *in-vitro* drug-drug interactions, and factors influencing clinical outcomes of CYP450 inhibition. This is expected to help researchers correctly identify CYP450 inhibitors as Friends or Foes in various discovery and clinical settings.

## Graphical Abstract

**Figure:**
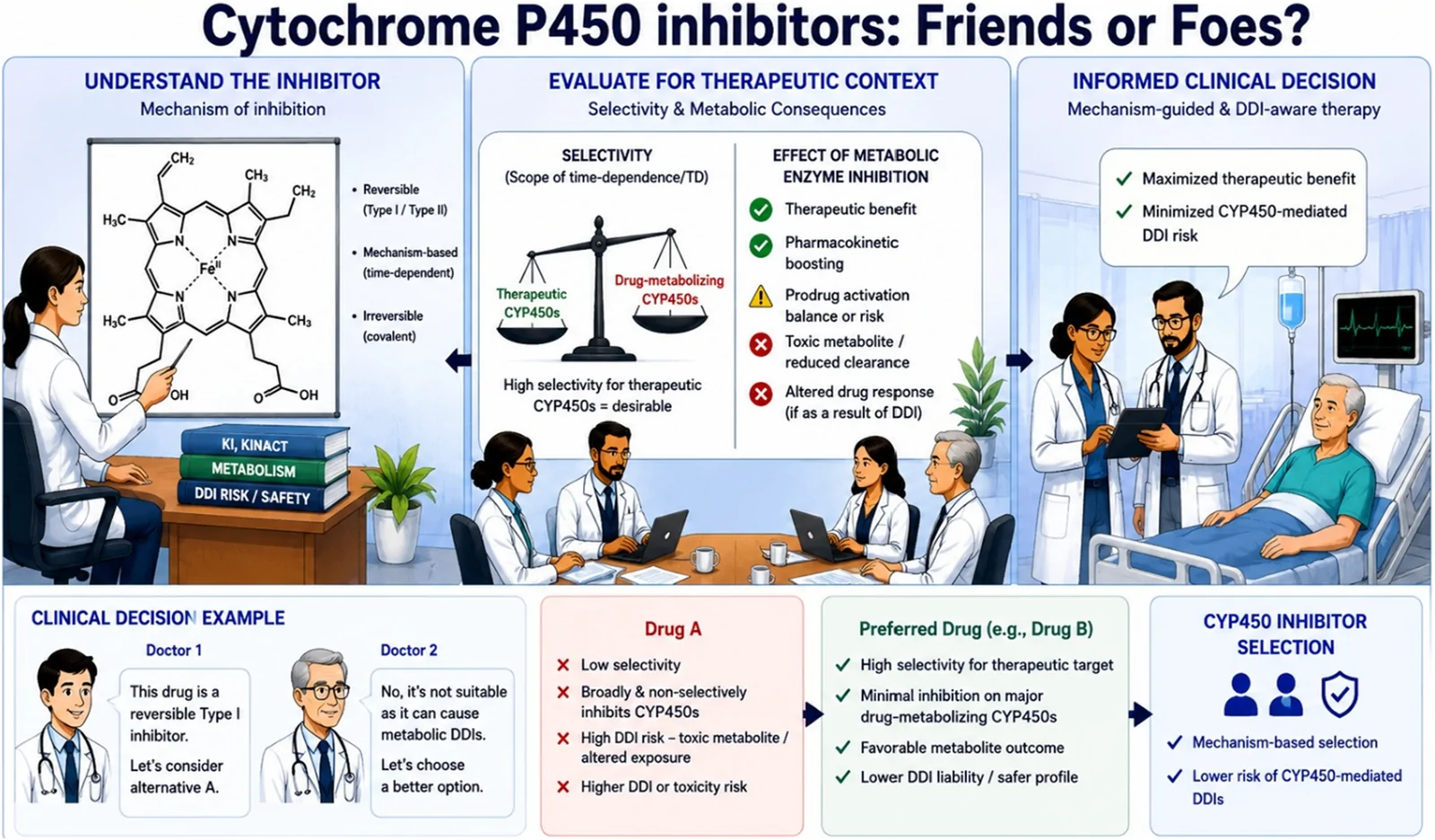

## 1 Introduction

Cytochrome P450 (CYP450) enzymes constitute a superfamily of heme-containing monooxygenases that play a central role in the metabolism of xenobiotics and endogenous compounds (Guengerich, 2015). These enzymes catalyse a wide range of oxidative transformations, including hydroxylation, epoxidation, and dealkylation, thereby facilitating drug clearance and maintaining physiological homeostasis. It is estimated that approximately 70%–80% of clinically used drugs are metabolised by CYP450 enzymes (see Figure 1), with major contributions from CYP3A4, CYP2D6, CYP2C9, CYP2C19, and CYP1A2 (Rendic and Guengerich, 2015). Inhibition of CYP450 enzymes has long been recognised as a critical factor influencing drug pharmacokinetics, efficacy, and safety. CYP450 enzyme inhibition is viewed as an undesirable property in drug discovery, primarily due to its association with drug–drug interactions (DDIs), metabolic instability, and toxicity (Guengerich, 2022). Consequently, regulatory agencies such as the U.S. Food and Drug Administration mandate systematic evaluation of CYP450 inhibition and induction potential during preclinical and clinical development to minimise adverse outcomes.

**FIGURE 1:**
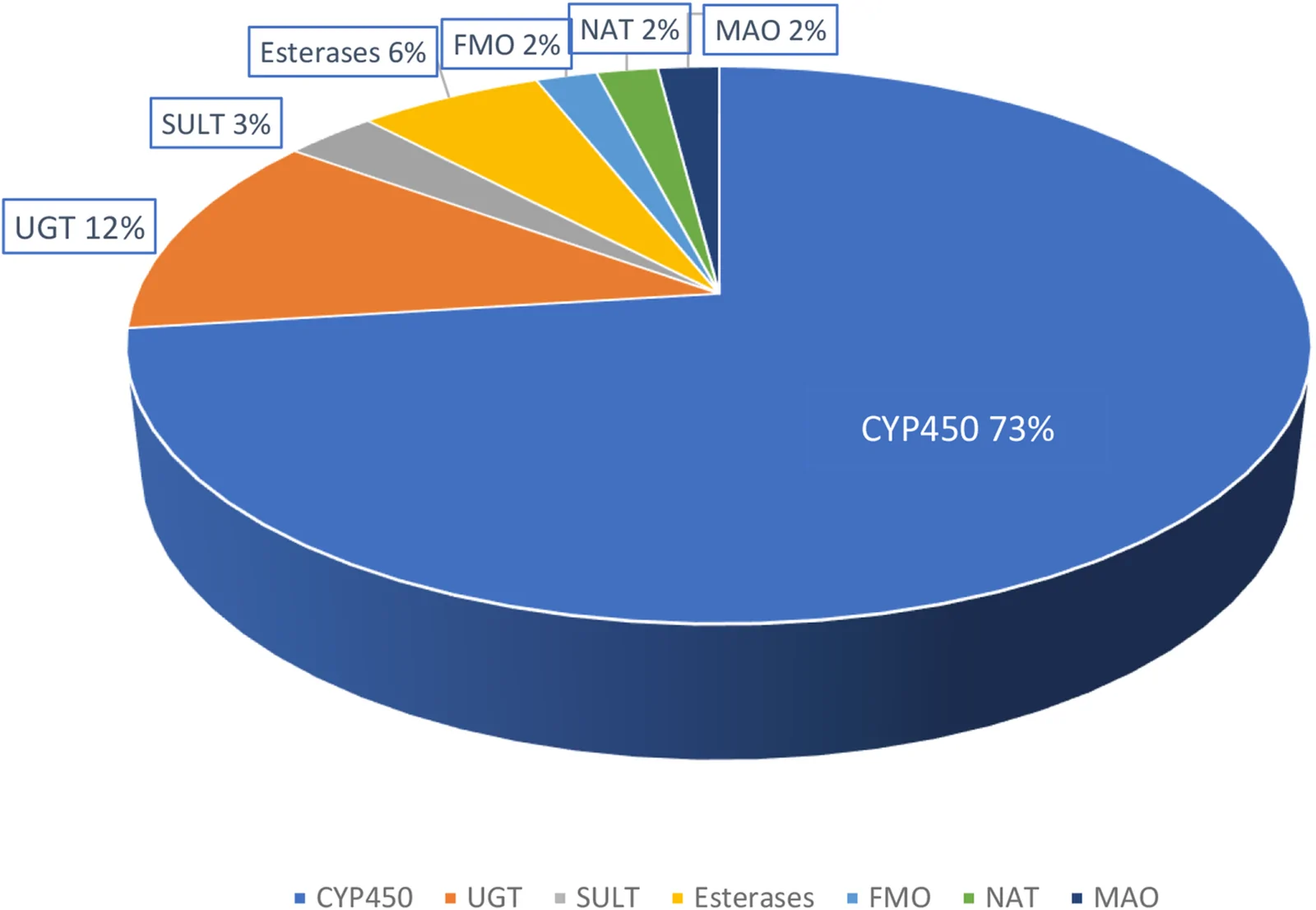
Percentage contribution of different drug metabolising enzymes (Laurence et al., 2011). In these enzymes, CYP450 has a major (73%) contribution, while others have a total of 27% but significant contribution to drug metabolism.

However, CYP450 inhibition is not inherently detrimental and can be fine-tuned to provide therapeutic benefits (see Figure 2). Selective inhibition of specific CYP450 isoforms has been successfully exploited as a therapeutic strategy in multiple disease areas, including hormone-dependent cancers and infectious diseases. As reported earlier, certain CYP450 isoforms are valuable drug targets, particularly when inhibition modulates key biosynthetic pathways (e.g., CYP19A1 catalyses the conversion of androgens to estrogens, and its inhibition by letrozole and anastrozole reduces estrogen levels, thereby limiting the proliferation of hormone-dependent breast cancer cells (Miller, 2003). Similarly, CYP17A1 plays a critical role in androgen biosynthesis, and its inhibition by abiraterone suppresses androgen production, providing therapeutic benefit in prostate cancer (Attard et al., 2009). In endocrine regulation, CYP11B1 and CYP11B2 are involved in corticosteroid and aldosterone biosynthesis, and their inhibition has been explored for treating disorders such as Cushing’s syndrome and hypertension (Papillon et al., 2015). In infectious diseases, fungal CYP51 catalyses the demethylation of lanosterol, a key step in ergosterol biosynthesis. Inhibition of this enzyme by azole antifungals such as ketoconazole, itraconazole, and fluconazole leads to depletion of ergosterol and accumulation of toxic sterol intermediates, ultimately disrupting fungal cell membrane integrity and viability (Odds et al., 2003). Likewise, CYP450 mediated pathways in pathogens have been explored as therapeutic targets in diseases such as tuberculosis and leishmaniasis, where inhibition of essential metabolic enzymes compromises pathogen survival (Ouellet et al., 2010). These examples clearly illustrate that CYP450 inhibition can be strategically exploited as a pharmacological “friend” when directed toward disease-relevant pathways.

**FIGURE 2:**
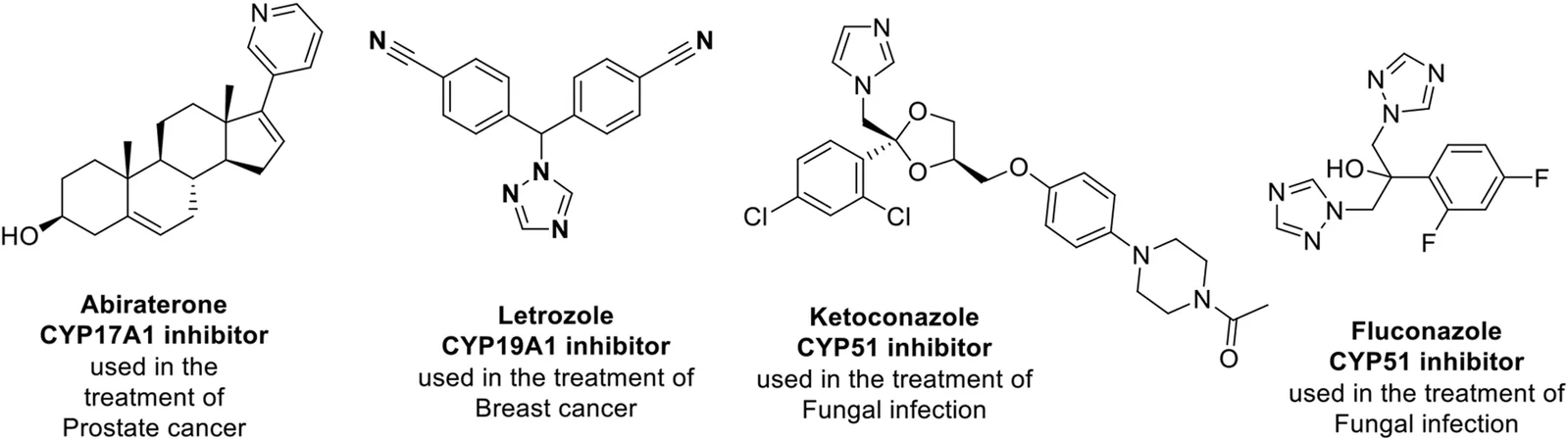
Representative examples of CYP450 inhibitors used as drugs in the treatment of cancer and the fungal infection.

However, the same inhibitory interactions can also lead to undesirable clinical outcomes. Non-selective or unintended CYP450 inhibition disrupts normal drug metabolism, resulting in altered pharmacokinetics, drug-drug interactions, and toxicity due to increased systemic exposure of co-administered drugs. A classic example is the inhibition of CYP3A4 by ketoconazole, which significantly increases plasma levels of drugs such as midazolam and simvastatin, leading to excessive sedation or risk of rhabdomyolysis respectively (Greenblatt et al., 2011). Similarly, inhibition of CYP2C9 by fluconazole can elevate warfarin levels, increasing the risk of bleeding complications (Gericke, 1993). These clinically significant interactions highlight how CYP450 inhibition can act as a pharmacological “foe” (by altering drug pharmacokinetics and inducing off-target effects) when selectivity and mechanistic understanding are lacking. Several prodrugs require CYP450-mediated metabolism to generate their active forms, and inhibition of these pathways can lead to therapeutic failure. Clopidogrel is the prodrug and requires activation by CYP2C19, and co-administration with CYP450 inhibitors such as omeprazole reduces its active metabolite formation. This diminishes antiplatelet efficacy and increases cardiovascular risk. These examples illustrate that CYP450 inhibition not only affects drug clearance but can also alter pharmacological outcomes depending on the metabolic role of the enzyme.

Selective CYP450 inhibition is both a significant challenge and therapeutic opportunity for novel drug discovery. The ability to inhibit specific CYP450 isoforms can be of therapeutic value, but unintended CYP450 inhibition is a cause of pharmacokinetic (PK) variability, drug–drug interactions (DDIs) and drug attrition during drug development (Guengerich, 2008; Zanger and Schwab, 2013). There are several mechanisms of CYP450 inhibition (Denisov et al., 2005; Guengerich, 2015), each of which plays a particular role in metabolic activity. Although traditionally CYP450 inhibition has been considered as a “bad thing,” it has been effectively used in some therapeutic areas such as cancer, fungal and viral infections. Importantly, the ultimate therapeutic gain or side effects of any CYP450 inhibitor are expected to be linked to several interconnected factors related to its selectivity for a particular isoform, the mechanism of inhibition, its metabolic stability, its tissue distribution, its binding affinity, and its capacity to generate reactive intermediates. Yet, even with the significant progress made in the understanding of CYP450 inhibition, it can be difficult to determine when it will be clinically useful and when it will trigger toxicity and/or clinically relevant DDIs (Rendic and Guengerich, 2015). This complexity remains a significant hurdle in the rational design and drug safety assessment, as well as lead optimisation.

In this review, the structural, mechanistic and medicinal chemistry aspects of cytochrome P450 inhibition are discussed. The catalytic cycle, structural dynamics, and mechanism of CYP450 inhibition are very complicated, nonetheless well understood in the literature (Dubey and Shaik, 2019; Kalita et al., 2020; Dixit et al., 2022). These usually involve reversible and irreversible often called mechanism-based inhibition mechanisms. Nonetheless, there is some confusion in the literature about the nomenclature and classification of different CYP450 inhibition mechanisms and inhibitors.

There are different types of classifications reported in the literature, which have some overlap but also major differences and inconsistencies. CYP450 inhibitors are traditionally classified according to their biochemical mechanisms, including reversible inhibitors, irreversible inhibitors, metabolite-intermediate complex (MIC) formers, mechanism-based inhibitors, and slow or tight-binding inhibitors (Guengerich, 2022). However, these schemes often do not fully align with the US-FDA framework/guidelines for evaluating the CYP450 inhibition potential of candidate drugs. Moreover, this literature classification frequently overlooks the distinction between Type I and Type II ligands while classifying CYP450 inhibitors. This causes overlapping terminology and potential confusion for students, researchers, and general readers. To provide a more consistent and regulatory-relevant perspective, a revised classification is presented below, which organizes CYP450 inhibitors into the two major categories of reversible and mechanism-based (time-dependent) inhibitors, while incorporating the spectroscopic classification of Type I and Type II binding interactions (see Figure 3).

**FIGURE 3:**
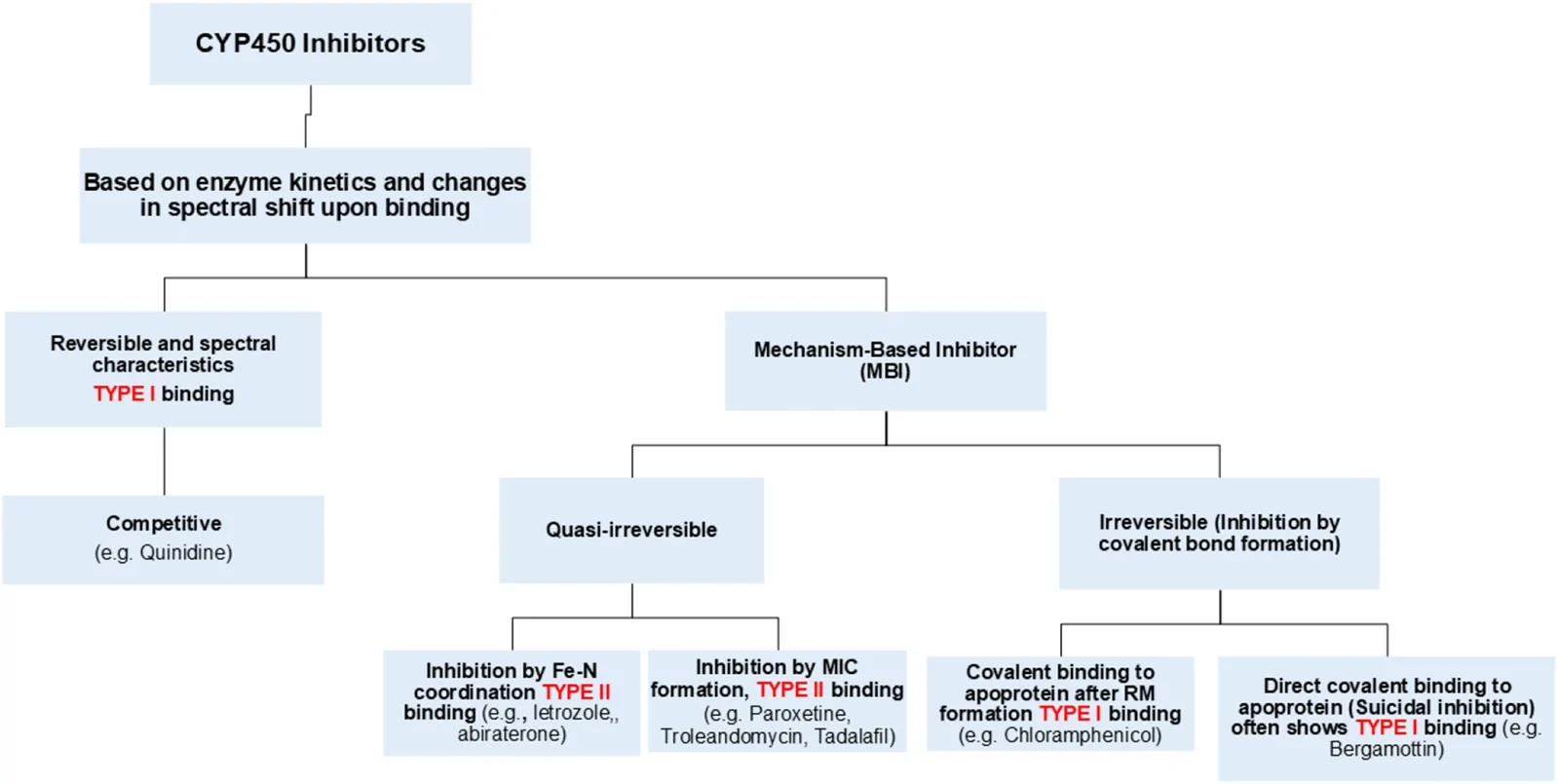
Combined classification of the CYP450 inhibitors based on the mechanism of inhibition and spectral pattern. CYP450 inhibitors are classified as reversible and mechanism-based inhibitors. The reversible inhibitors usually have a competitive mechanism and show type I binding (spectrum, with an increase in absorbance near 385–390 nm and a corresponding decrease near 418–425 nm) while interacting with the active-site residue. MBIs classified into two subclass quasi-irreversible and irreversible. The quasi-irreversible inhibitors demonstrate type II spectral features while inhibiting CYP450 by metabolite intermediate complex (MIC) formation (absorption bands with a maximum typically observed between 425 and 435 nm and a minimum near 390–410 nm) while interacting with the Heme-Fe porphyrin ring. Irreversible inhibitors also show type I binding since they do not form coordinate bonds with Heme-Fe and bind directly or after reactive metabolite formation to the apoprotein.

In Section 5 a detailed and systematic overview of the *in vitro* approaches recommended by FDA guidelines for investigating CYP450 substrates, inhibitors, and inducers, along with a decision tree for their evaluation, is presented. The different factors that help predict the beneficial or detrimental outcomes of CYP450 inhibition are also discussed in Section 6. In addition, the review summarises recent CYP450 inhibitor prediction models developed over the past 5 years using diverse approaches, including artificial intelligence (AI), machine learning (ML), deep learning, and large language models (LLMs) in the last section. Together, this review provide a comprehensive understanding of CYP450 inhibition, proposes a modified classification scheme, protocol, and highlight the recent advances, applications, and limitations of different models developed in the last 5 years.

## 2 Cytochrome P450 catalytic cycle and enzymatic mechanisms

The CYP450 catalytic cycle provides the mechanistic foundation for understanding both substrate oxidation and the diverse modes by which inhibitors modulate enzyme activity. This multistep process couple’s substrate binding to sequential electron transfer, molecular oxygen activation, and proton transfer, ultimately generating the highly reactive ferryl intermediate, Compound I, which catalyses substrate oxidation (Denisov et al., 2005; Guengerich, 2018). Importantly, each stage of the catalytic cycle represents a potential mechanistic checkpoint at which substrate binding, inhibitor binding, or disruption of electron transfer can influence CYP450 activity.

The CYP450 catalytic cycle (Shaik et al., 2010) starts from a resting state containing iron in the ferric [Fe(III), d^5^] form (see Figure 4) with coordinating water. Thus, the resting enzyme state exhibits a relatively low reduction potential and is resistant to electron transfer. In the first (1^st^) step, binding of a substrate displaces the coordinated water molecule, converting the heme into a pentacoordinate high-spin Fe(III) (S = 5/2) state with an increase in the heme reduction potential by 130–300 mV. This induces the first electron transfer (second step) from NADPH-CYP450 reductase (Denisov et al., 2005). The third step involves oxygen binding forms followed by second electron transfer (fourth step) and proton transfer (fifth step) generating ferric-peroxo and hydroperoxo (Compound 0) intermediates respectively.

**FIGURE 4:**
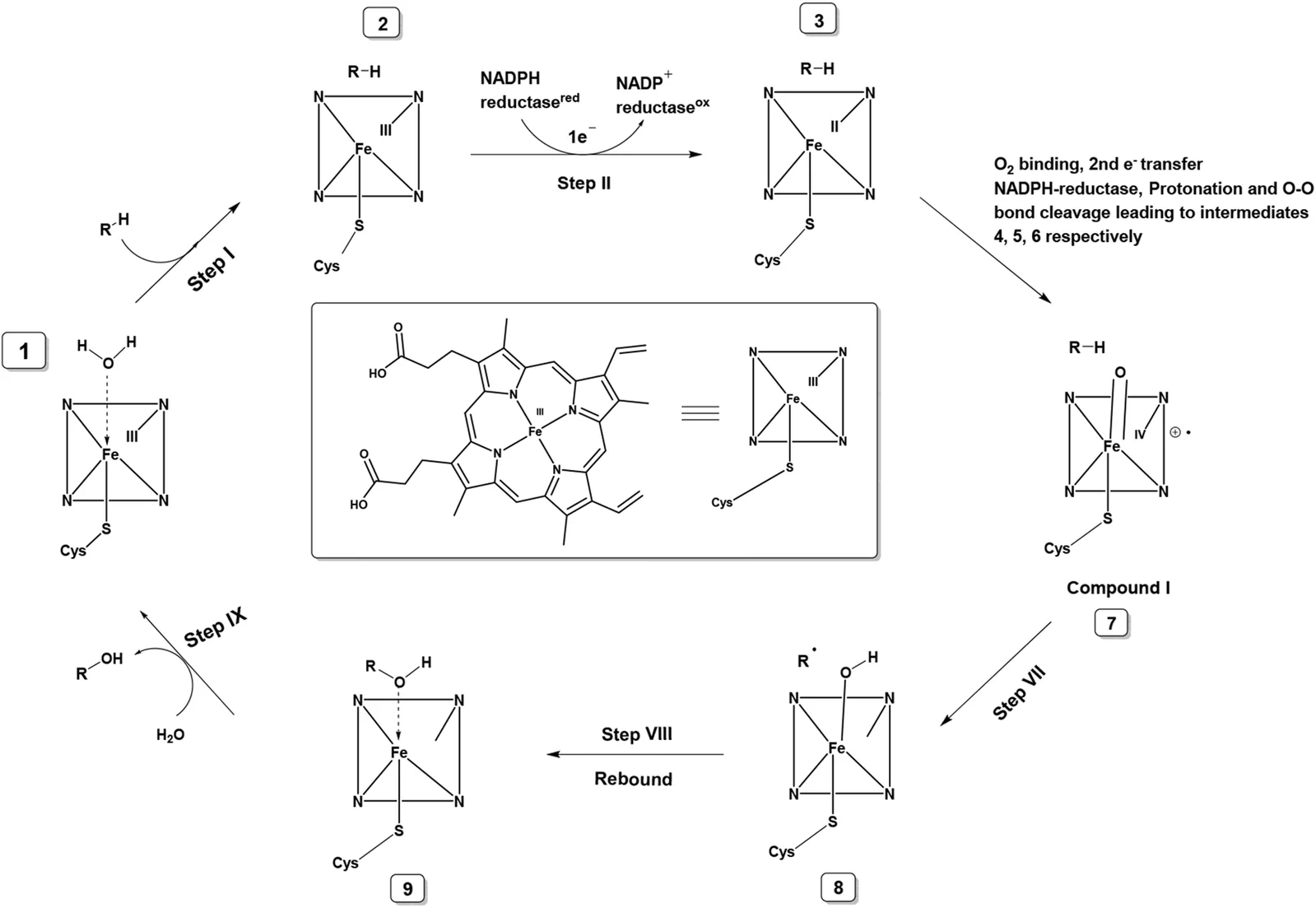
The catalytic cycle of CYP450 involves substrate binding, electron transfer, oxygen activation, formation of the reactive ferryl-oxo intermediate (Compound I), substrate oxidation, release of the product, and regeneration of the resting-state ferric enzyme (Shaik et al., 2010). The highlighted heme prosthetic group represents the principal site of substrate binding and CYP450 inhibition.

The sixth step produces, Cpd-I, a highly reactive oxidising intermediate responsible for the characteristic oxidative chemistry of CYP450 enzymes. Its highly electrophilic nature enables the seventh step, hydrogen abstraction, oxygen-atom transfer, epoxidation, and heteroatom oxidation of a broad range of substrates. Subsequent, eighth step, radical rebound results in the formation of the oxidised product (R-OH), which dissociates from the active site regenerating the resting state to continue the catalytic cycle. This sequence of substrate binding-induced electron transfer, oxygen activation, hydrogen abstraction by Cpd-I, and radical rebound is an important determinant of the regio- and chemo selectivity of CYP450-mediated substrate oxidation (Guengerich, 2018).

Substrate-like molecules, which are typically Type I ligands, displace the distal water ligand and promote the low-spin to high-spin transition, thereby enhancing the electron transfer rate. Ligand binding can also modulate the catalytic cycle through direct heme coordination. These Heme-Fe coordinating ligands, called as Type II ligands, are mostly heterocycles containing sp^2^-hybridised nitrogen such as imidazole, triazole, or pyridine, which coordinate directly to the ferric heme iron through the lone pair on the heteroatom. This interaction restores the ligand field, stabilises the low-spin state, suppresses reduction of the heme iron, and consequently inhibits oxygen activation and catalysis (Guengerich, 2015; Deodhar et al., 2020).

Often, when the catalytic cycle progresses, certain substrates undergo CYP450-mediated bioactivation to generate electrophilic metabolites that covalently modify either the heme prosthetic group or nucleophilic amino acid residues within the apoprotein, producing mechanism-based (suicide) inhibition that permanently inactivates the enzyme (Deodhar, et al., 2020). Additionally, other electron transfer uncoupling reactions can be triggered in the absence of substrate ligands and reduce catalytic efficiency while contributing to oxidative stress and cellular toxicity (Baylon et al., 2013). These factors that modulate the CYP450 catalytic cycle and provide the molecular basis for rational inhibitor design, prediction of drug–drug interactions, and the therapeutic exploitation of CYP450 inhibition are discussed in Section 3.

## 3 CYP450 inhibition, types, mechanisms and spectral patterns

The catalytic cycle of CYP450 enzymes includes several critical steps at which substrate metabolism can be inhibited (see Figure 4). Among these, substrate binding and the first electron transfers are particularly susceptible to inhibition since these are often the rate-determining steps. Moreover, the CYP450 catalytic cycle, mechanisms of substrate oxidation, inhibition, spectroscopic features, and the impact of drug metabolism on pharmacological and clinical responses have been studied over the years by researchers from a wider spectrum of the scientific community. These include chemists, biochemists, crystallographers, analytical chemists, pharmacologists, drug metabolism scientists, clinicians, regulators and even patients. This has resulted in the use of overlapping terminologies in different literature sources, which often leads to inconsistent descriptions of CYP450 inhibitor classification. Therefore, here, we provide a potentially improved classification framework or approach which captures the main features of CYP450 inhibitors studied in different fields.

In this unified approach shown in Figure 3, CYP450 inhibitors are classified into two major categories: (i) Reversible and (ii) Mechanism-based inhibitors (MBIs, also called time-dependent inhibitors). The reversible inhibitors mostly have a competitive mechanism and exhibit Type-I spectroscopic features (discussed in Section 4), whereas the MBIs are classified into irreversible and quasi-irreversible types and may demonstrate both Type-I or II spectroscopic features (see Figure 3). MBIs often require metabolic activation, which progressively reduces enzyme activity during incubation. These are further divided into 1) Quasi-irreversible inhibitors, which cause inhibition through nitrogen atom coordination or through forming stable metabolite–intermediate complexes (MICs) with the heme iron, and 2) irreversible inhibitors, which permanently inactivate the enzyme either through direct covalent or reactive metabolite bonding with heme-porphyrin or active site residues. In the following sections, various CYP450 inhibitors and their kinetic parameters (e.g., *V*_max_ *K*_m_, *k*_inact_ and *K*_obs_), spectral characteristics, and inhibitor pharmacophoric features are discussed in detail.

### 3.1 Reversible inhibitors compete with substrate but do not undergo oxidation or inhibit the CYP450 catalytic cycle

CYP450 inhibitors that compete with substrates reversibly for the CYP450 active site but do not cause permanent inhibition are classified as competitive inhibitors. Due to this competition, the substrate’s access to the active site is hindered. As the inhibitors do not cause a sufficient rise in the redox potential essential for the first electron transfer, the catalytic cycle is inhibited. These molecules also have (a) hydrophobic, and/or (b) specific hydrogen bonding or ionic interactions with active-site residues. Competitive inhibitors are usually structurally similar to the endogenous substrate or xenobiotics and thus share the substrate-binding site. CYP450 isoforms possessing relatively constrained active sites, such as CYP2D6 and CYP2A1, may exhibit classical competitive inhibition kinetics because ligand binding occurs within a more structurally defined substrate-binding pocket.

The reversible inhibitor occupies the catalytic site, making substrate binding less favorable and reducing the apparent affinity of the substrate. This binding is studied using the Michaels-Menten formalism; ( E+I⇌EI ) and K_i_ = *k*_off_/*k*_on_, where K_i_ is the equilibrium dissociation constant (also called the inhibition constant), and *k*_off_ and *k*_on_ are dissociation and association rate constants. This model for reversible inhibition involves a term $\alpha =\left(1+\frac{I}{K_{i}}\right)$ called the inhibition factor and reflects the extent of inhibition: higher inhibitor concentration $\left[I\right]$ increases inhibition, lower $K_{i}$ indicates stronger inhibitor binding. Therefore, the apparent $K_{m}$ increases, and higher substrate concentrations are required to achieve the same reaction rates, but $V_{\max}$ remains unchanged since higher substrate concentrations can outcompete the inhibitor.

$$V_{0}=\frac{V_{\max}\;\left[S\right]}{{\alpha K}_{m}+\left[S\right]}$$

Quinidine is a competitive and highly potent inhibitor of CYP2D6 and is commonly used in experiments to study CYP2D6 metabolism (see Figure 5) (McLaughlin et al., 2005). CYP450 enzymes seem to be unique since, despite large active sites and protein size which can support allosteric binding, the uncompetitive and mixed (non-competitive) inhibitions are very rarely reported in the literature (see (Shinkyo and Guengerich, 2011) for cholesterol-nifedipine example). However, this observation is most likely to be caused by selection and measurement bias, since the mechanism of the reversible inhibition is rarely studied further, and the majority of groups are focused on classifying the test compounds as either reversible or mechanism-based inhibitors. Thus, the experiments necessary to further elucidate mechanisms of reversible inhibition (namely,: competitive, uncompetitive, and mixed/non-competitive) are rarely conducted. This is a classic case of famous quote by Carl Sagan; “Absence of evidence is not the evidence of absence”.

**FIGURE 5:**
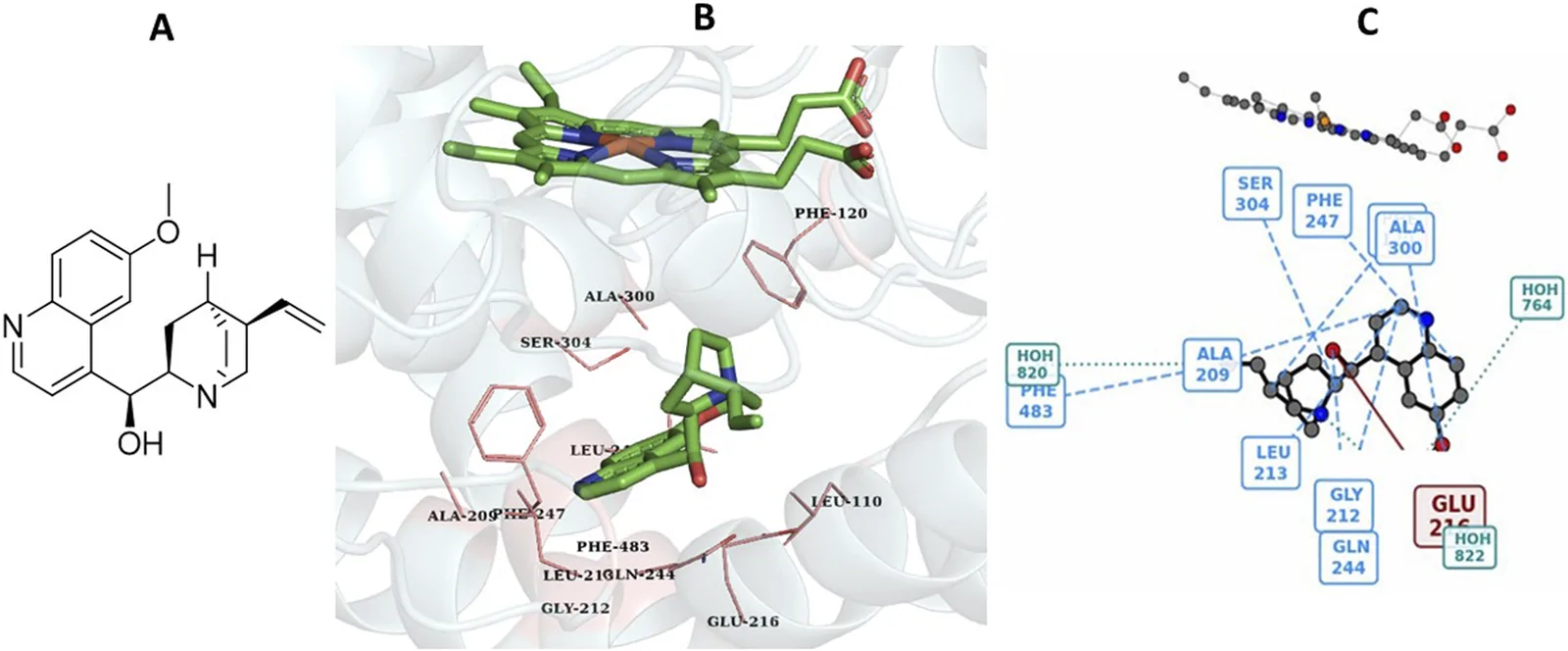
Binding mode and interaction analysis for a reversible inhibitor (quinidine; QDN) bound in the CYP2D6 active site (PDB ID: 4WNU). **(A)** 2D structure of quinidine. **(B)** 3D binding pose of quinidine within the active site, highlighting its interactions with the key residues LEU213, ALA209, GLU216, and PHE247; the heme group is shown with the central iron (Fe) atom in orange. **(C)** 2D interaction diagram of the quinidine bound in the active site showing the residues and water molecules contributing to ligand recognition. Blue dashed lines represent hydrophobic contacts, green dotted lines represent hydrogen-bond interactions, and the red line indicates a polar interaction involving GLU216. The 2D interaction diagram is built using an in-house tool available on GitHub.

For a conventional reversible inhibitor, the relationship between inhibitor potency (IC_50_) of the CYP450 inhibitors and inhibition constant (Kᵢ) is commonly described by the Cheng-Prusoff equation (Burlingham and Widlanski, 2003):

$$K_{i}=\frac{{IC}_{50}}{1+\frac{\left[S\right]}{K_{m}}}$$

where ([S]) is the substrate concentration and (*K*_m_) is the Michaelis–Menten constant of the probe substrate (Yung-Chi and Prusoff, 1973). This equation assumes that the substrate and inhibitor are distinct molecules competing for the same enzyme active site and that the inhibitor does not undergo catalysis.

Under these conditions, substrate and inhibitor concentrations are independent variables, allowing K- to be estimated directly from IC_50_ measurements. Representative CYP450 inhibitors are Mifepristone (Du et al., 2019), Danazol (Lee et al., 2012), and Diltiazem (Sutton et al., 1997) (see Figure 6; Table 1).

**FIGURE 6:**
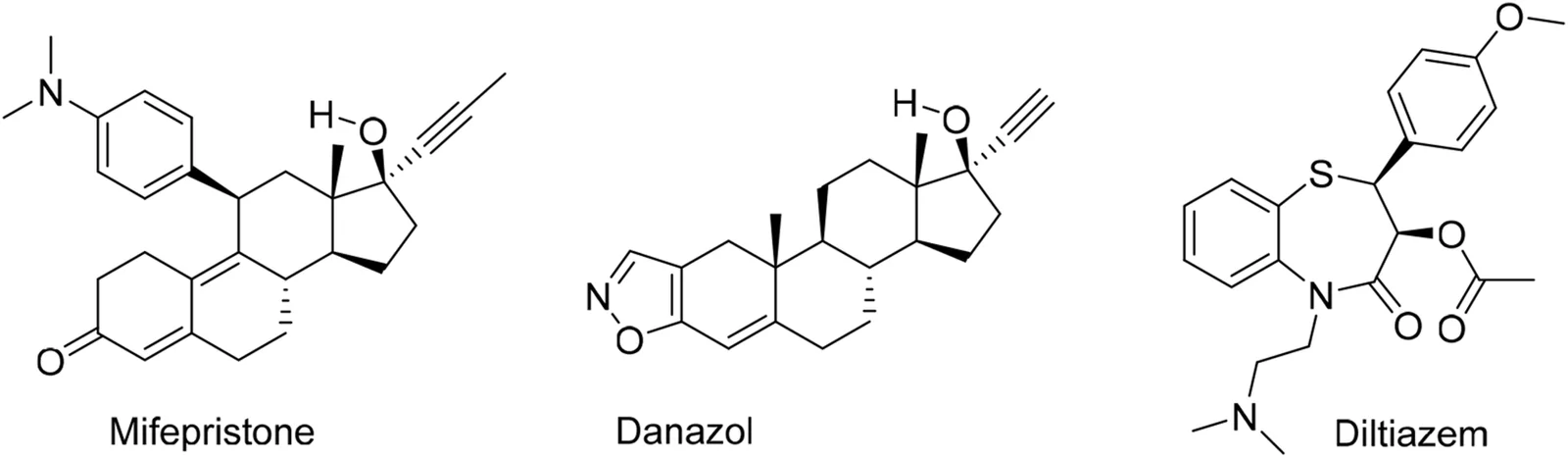
Other examples of true reversible inhibitors (besides quinidine) that inhibit a CYP450 isoform without being metabolised by the same isoform (see Table 1; Figure 5).

**TABLE 1:** Examples of reversible (Type I) CYP450 inhibitors with their kinetic parameters. These inhibitors demonstrated their mechanism of inhibition by displacing the bound ligand from the active site without disrupting the catalytic cycle. These molecules are true inhibitors that are not metabolised by the same isoform. For Danzol *K*_m_ = 0.2 µM and for Diltiazem *K*_m_. = 1 µM.

Drug/Reference	CYP450 isoform	IC_50_ (nM)
Mifepristone	CYP3A4	9,500
Quinidine	CYP2D6	10
Danazol	CYP2J2	77
Diltiazem	CYP3A4	120,000

However, this assumption (that substrate and inhibitor are distinct molecules) is no longer valid when a compound acts as both a substrate and a reversible inhibitor of the same CYP450 isoform. In such cases, the same molecule can bind the enzyme in two different conformational states. In one conformation, the molecule undergoes metabolism, while in the second, it causes competitive inhibition. Consequently, substrate and inhibitor concentrations are intrinsically linked, and the classical Cheng-Prusoff relationship cannot adequately describe the system. Instead, the enzyme kinetics must be represented by two competing binding equilibria: an enzyme–substrate complex characterized by *K*_m_ and a non-productive inhibitory complex characterized by *K*_i_. Assuming rapid equilibrium, the reaction velocity can be expressed as.

Reaction velocity calculation for the molecule which act as a substrate in the one confirmation and also act as a inhibitor in second confirmation (Equation 1).

$$v=\frac{V_{\max}\frac{S}{K_{m}}}{1+\frac{S}{K_{m}}+\frac{S}{K_{i}}}$$ (1)

Multiplying the numerator and denominator by *K*_m_ gives;

$$v=\frac{V_{\max}\;S}{K_{m}+S\left(1+\beta \right)}$$

where (S) is the concentration of the dual substrate–inhibitor and *β* = *K*_m_/*K*_i_. This expression assumes that the substrate concentration is kept near the K_m_ value during the experiment. It demonstrates that the ratio of *K*_m_ and *K*_i_ *(β*) governs the balance between metabolism and inhibition. When (β > 10 i.e., log *β* > 1), the enzyme increasingly forms non-productive complexes, resulting in substrate self-inhibition and a reduction in the attainable metabolic rate. Conversely, when (log *β* < 1), productive binding predominates, and the system approaches classical Michaelis–Menten kinetics. When log *β* = 1, even under saturating substrate (which is also an inhibitor) conditions, the effective reaction velocity cannot reach V_max_/2, indicating that half of the enzyme molecules may be bound in unproductive (inhibitory) conformations. Consequently, the log of dimensionless ratio, log *β* = log(*K*_m_/*K*_i_), may serve as a useful tool to study substrate–inhibitor balance, with values less than unity indicating predominantly substrate-like behavior and values greater than unity indicating dominating inhibitory behavior. Since *IC*_50_ values are more readily available *β* = *K*_m_/*K*_i_ may be rewritten as 2 *K*_m_/*IC*_*50*_. For Phenacetin (Venkatakrishnan, 1998), (see Table 2; Figure 7) the reported values ( $K_{m}=9000$ nM, *IC*_*50*_ = 41 nM) yield a log *β* of approximately 2.64, indicating that inhibitory binding occurs at concentrations far below those required to achieve half-maximal catalytic turnover. Thus, the biochemical behavior of phenacetin indicates that the inhibitory conformation is strongly favored; however, the molecule can occasionally adopt a catalytically productive conformation within the CYP1A2 active site. A similar trend is observed for Omeprazole (Karam et al., 1996), where log β ≈ 1.07, suggesting a moderate preference for inhibitory binding relative to substrate metabolism. Clozapine (Fischer et al., 1992) shows log β = 0.44 indicating that the concentrations required for inhibition and metabolism remain comparable, consistent with a balanced substrate–inhibitor reactions at the active site. For, Dextromethorphan (Kerry et al., 1994) log β ≈ 0.19, implying that catalytic turnover becomes significant at concentrations comparable to those required for measurable inhibition. Such compounds behave predominantly as substrates, with metabolism competing effectively against inhibitory occupancy.

**TABLE 2:** Examples of reversible CYP450 Type I inhibitors which also act as substrates to varying extent, shown with their kinetic parameters. These inhibitors displace the bound ligand in the active site without disrupting the catalytic cycle. The calculation of parameter log β is valid only when the experiments are performed at [S] = K_m_.

Drug	CYP450 isoform	IC_50_ (nM)	*K*_m_ (nM)	β =2 * *K*_m_/*IC*_50_	Log β
Phenacetin	CYP1A2	41	9,000	440	2.64
Omeprazole	CYP2C19	2080	12,340	11.9	1.07
Clozapine	CYP2D6	18,000	25,300	2.8	0.44
Dextromethorphan	CYP2D6	1800	1,400	1.56	0.19

**FIGURE 7:**
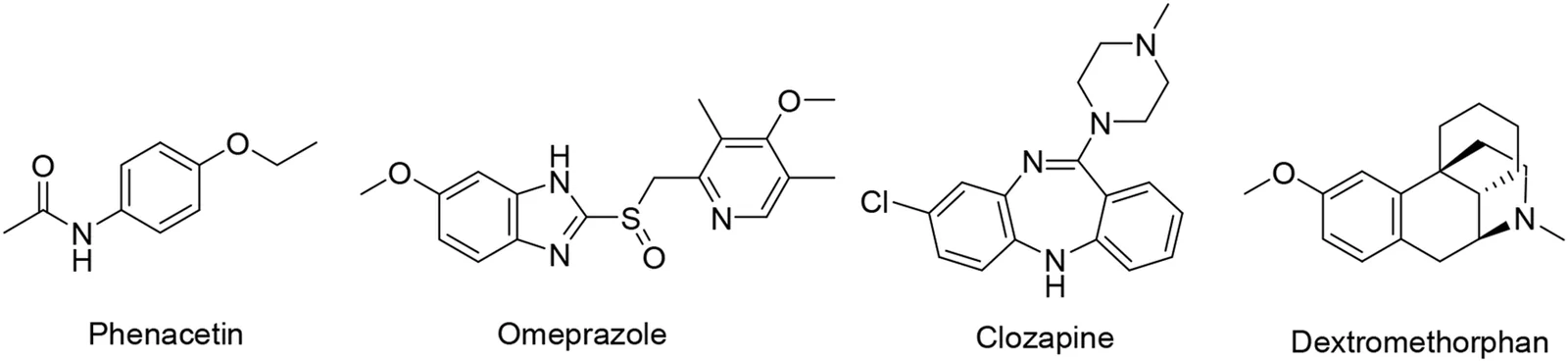
Example of reversible inhibitors that inhibit and are also metabolised by the same CYP450 isoform.

In the examples presented here, the trend in log *β* for Phenacetin > Omeprazole > Clozapine > Dextromethorphan reflects a shift from inhibitor-like to substrate-like interactions with respective CYP450 isoforms.

### 3.2 Mechanism-based inhibitors (MBI) or time-dependent inhibitors

In contrast to reversible inhibitors, whose inhibitory effects are rapidly lost upon dissociation from the active site, mechanism-based inhibitors (MBIs) or time-dependent inhibitors (TDIs) usually form irreversible complexes. They exhibit progressively increasing inhibition as a function of inhibitor concentration and preincubation time since the enzyme is gradually inactivated (Orr et al., 2012). MBI can occur if; 1) the inhibitor has a heterocyclic nitrogen that strongly coordinates with heme-Fe and inhibits the catalytic cycle, or 2) the reactive metabolites (RM) formed from the metabolism of the inhibitor bind covalently with amino acids or heme-porphyrin ring (often called suicidal inhibition). This irreversible binding is modelled using a two-step reaction involving 1) reversible binding and 2) irreversible (coordinate or covalent binding) with heme or active site residues. These irreversible inhibitors are characterised biochemically using two kinetic parameters, namely,; *K*_I_ and *k*_inact_ (see Equation 3 below) (Heppner et al., 2024). The *K*_I_ is the apparent inhibition constant measured from the *in-vitro* incubation experiments (discussed in detail below), and *k*_inact_ is the rate of irreversible binding (heteroatom-Fe, or electrophilic reaction with heme-porphyrin or nucleophilic amino acid side chains).

Two-step mechanism of Mechanism-based enzyme inhibition (Equation 2)

E+I⇌E.I⟶ E−I (2)

Where E = CYP450 enzyme, I = inhibitor, EI = reversible enzyme–inhibitor complex and E–I represents the irreversibly inactivated enzyme bound to the inhibitor.

Inactivation constant for irreversible inhibition (Equation 3).

$$K_{I}=\left(k_{off}+k_{inact}\right)/k_{on}$$ (3)

This equation can be rearranged into the following form.

Partitioning ratio for covalent inhibitors that is a measure of the contributions of reversible and covalent/coordinate binding (Equation 4).

$$K_{I}=K_{i}*\left(1+k_{inact}*\frac{1}{k_{off}}\right)$$ (4)

$$\frac{k_{inact}}{k_{off}}=\left(\frac{K_{I}}{K_{i}}-1\right)$$

The above equation is useful since it relates two equilibrium constants with the rate constant for covalent/coordinate binding and dissociation rate constant. This reformulation converts an equation that required very difficult-to-measure parameters (*k*_off_ and *k*_on_) into relatively easily measurable equilibrium constants and provides insights into the relative measure of reversible vs. irreversible binding via the partitioning ratio (*k*_inact_ */k*_off_). Thus, relative contribution of reversible (*K*_i_) and covalent binding (*k*_inact_) can be assessed and studied. When *k*_inact_ and *k*_off_ values are similar both reversible and irreversible (covalent/coordinate) binding contribute to the inhibition equally. The partitioning ratio can be estimated using Equation 4, i.e., from the ratio of the apparent inhibition constant (K_I_) to the reversible inhibition constant (K_i_). A larger value of the partitioning ratio indicates a predominantly faster irreversible binding mechanism, whereas values between 1 and two indicate balanced reversible binding (since 1 < K_I_/K_i_ < 2), and values between 1 and zero mean equal contribution of the two processes. Equation 4 requires K_I_ be greater than K_i_, thus negative values for partitioning ratio indicates either differences or errors in experimental protocols, and/or the breakdown of the basic assumption of the two-step binding process (Equation 2).

Another useful way to represent this relationship is *k*_inact_/*K*_I_ = *k*_on_ * *k*_inact_/(*k*_off_ + *k*_*inact*_) (Heppner et al., 2024). Since most irreversible inhibitors exhibit high *k*_inact_ values (very fast reactions), it is often not possible to estimate conventional IC_50_ or *K*_i_ parameters for irreversible inhibitors (see Tables 3, 4). A higher $k_{inact}/K_{I}$ value indicates that the inhibitor binds efficiently and rapidly inactivates the enzyme, whereas lower values reflect weak or inefficient inactivation. For a reversible inhibitor, Equation 4 simplifies to *K*_I_ = *K*_i_, since *k*_inact_ is zero and residence time should be estimated directly from the inverse of *k*_off_. For an inhibitor with mixed reversible and irreversible characteristics, *K*_i_ remains measurable, and preincubation also allows the measurement of K_I_ (see Table 4). Based on the mechanism of inhibition, the MBIs are also further divided into two subclasses: (1) Quasi-irreversible inhibitors, and (2) Irreversible inhibitors. These classes of MBIs are discussed below, including their key structural characteristics and kinetic properties (see Figure 3).

**TABLE 3:** Representative examples of CYP450 mechanism-based quasi-irreversible Type I inhibitors with their inhibition kinetic parameters. K_i_ values are estimated from IC_50_ values using the Cheng-Prusoff equation.

Drug	CYP450 isoform	IC_50_ (µM)	K_i_ (µM)	K_I_ (µM)	*k*_inact_ (min^-1^)	*k*_inact_/*k*_off_
Paroxetine	CYP2D6	2.54	1.27	4.85	0.17	2.82
Tadalafil	CYP3A4	22.6	11.3	12	0.21	0.06

**TABLE 4:** Examples of CYP450 mechanism-based irreversible inhibitors with their inhibition kinetics parameters. K_i_ values are estimated from IC_50_ values using Cheng-Prusoff equation.

Drug	CYP450 isoform	IC_50_ (µM)	*K*_*i*_ (µM)	*K*_I_ (µM)	*k*_inact_ (min^-1^)	*k*_inact_/*k*_off_
Furafylline	CYP1A2	0.07	23	23	0.87	0.0
Bergamottin	CYP3A4	2.42	7.7	22.4	0.087	1.9
Clopidogrel	CYP2C19	28.6	14.3	14.3	0.055	0.0
Binimetinib	CYP1A2	---	2.8	15.47	0.063	4.53
Mibefradil	CYP3A4	---	1	2.3	0.40	1.30
Zileuton	CYP1A2	---	50	117	0.035	1.34

#### 3.2.1 Quasi-irreversible inhibitors

The Quasi-irreversible inhibitors represent a class of MBIs that coordinate with heme iron. This coordination of heme iron is usually with a heterocyclic nitrogen of the ligand and occasionally with an RM (e.g., a carbene or nitrene, which is usually formed via oxidative metabolism). Since the inhibitory complex remains tightly associated with the enzyme for prolonged periods, enzyme activity may appear irreversibly lost under normal assay and pharmacological conditions. However, because the enzyme structure itself is not permanently damaged, activity can often be restored *in vitro*, thus getting the name “Quasi-irreversible”). Various reagents, irradiation at 440–500 nm, or oxidising to the ferric state using ferricyanide can disrupt the complex. Two types of mechanisms of quasi-irreversible inhibition have been identified: (i) Inhibition by Fe-N coordinate bond formation and (ii) Inhibition by metabolite intermediate complex (MIC) formation (see Figure 3) (Orr et al., 2012; Mirzaei et al., 2021). These two types are discussed briefly below.

##### 3.2.1.1 Inhibition by forming a coordination bond between the iron of the heme porphyrin ring and sp^2^ nitrogen (Fe-N) atom of the ligand

Direct coordination of heterocyclic sp^2^ nitrogen atoms to the iron of heme porphyrin ring in CYP450 represents an important mechanism of inhibition. sp^2^ nitrogen atom present in heterocyclic molecule like imidazole and triazole, can replace the axial water molecule by coordination to form a stable Fe←N bond. This coordination stabilizes the low-spin ferric heme state and produce Type II spectral response (details discussed in Section 4.2). A well-known example is miconazole (see Figure 8), in which the imidazole nitrogen atoms can coordinate with Heme-Fe and inhibit the CYP450 isoforms. Miconazole inhibits CYP2C9 with an *IC*_50_ of 2.0 µM (Ramsden et al., 2022) and a lower *K*_I_ of 15 μM, together with a faster irreversible binding rates *k*_inact_ = 0.21 min^-1^. Using Equation 4, these values give a partitioning ratio (*k*_inact_ */k*_off_) of 14, in agreement with the fact that irreversible binding is fast. Therefore, miconazole exhibits a pronounced quasi-irreversible inhibition of CYP2C9 by forming an Fe-N coordinate bond.

**FIGURE 8:**
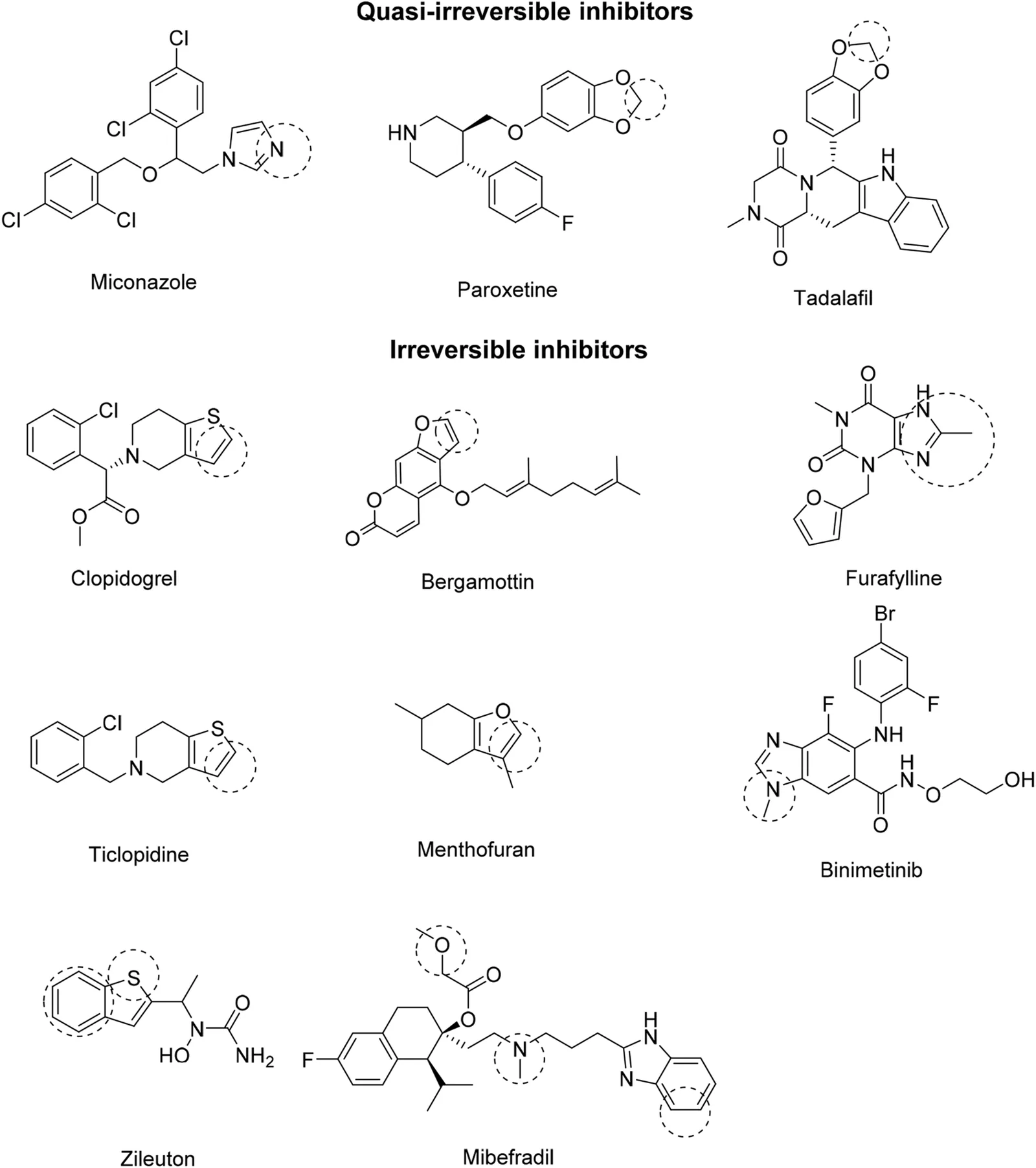
CYP450 inhibitors with quasi-irreversible, and irreversible which is also showed dual mechanism (reversible and irreversible) of inhibition. The atoms/groups inside dotted circle shows the site of metabolism (SOM), which form reactive metabolite intermediate complex (see Figure 3).

##### 3.2.1.2 Inhibition through metabolite intermediate complex (MIC) formation with Heme-Fe (nitroso and carbene complexes)

MIC complex formation can occur in two ways: 1) C-H oxidation at methylenedioxyphenyl (MDP) groups followed by formation of a reactive carbene intermediate which coordinates with Heme-Fe, and 2) in some drugs and drug-like compounds undergo sequential N-dealkylation and oxidation, generating a hydroxylamine intermediate that is further oxidised to a reactive nitroso species which coordinates with Heme-Fe to inhibit the catalytic cycle. The classic examples include paroxetine, tadalafil, and methylenedioxymethamphetamine, which produce characteristic carbene–heme complexes with CYP450 (Kamel and Harriman, 2013). Paroxetine, a quasi-irreversible inhibitor of CYP2D6, has a K_i_ = 1.27 µM, *K*_I_ = 4.85 µM, *k*_inact_ = 0.17 min^-1^ (Bertelsen et al., 2003). These give a partitioning ratio of 2.82, suggesting a faster irreversible binding of Paroxetine with CYP450.

Tadalafil a CYP3A4 inhibitor (see Table 3; Figure 8), has a partitioning ratio near zero (0.06) due to K_i_ = 11.3 µM, *K*_I_ = 12 μM, *k*_inact_ = 0.21 min^-1^ (Ring et al., 2005; Yu et al., 2024). Thus, Tadalafil has equal contributions from reversible and irreversible binding in its inhibitory potency. The methylenedioxyphenyl groups in paroxetine and tadalafil are oxidized to a reactive carbene species which forms carbene-heme complex at approximately same rate. This suggests that the rate of the reaction is mostly governed by structure of the reactive group and corresponding reaction mechanism, whereas the binding affinity is a function of the full structure of the molecules. The second category of quasi-irreversible MIC-type inhibitors include compounds containing alkylamine functionalities, particularly tertiary amines. These form nitroso complexes which block oxygen binding and the catalytic cycle, thereby making the enzyme catalytically inactive. Examples include troleandomycin and erythromycin, whose tertiary amine-containing amino sugar moieties are metabolically activated to nitroso intermediates that bind to CYP3A4. The MICs of troleandomycin are spectroscopically detectable by an absorbance peak near 455 nm. Similar mechanisms have also been reported for clorgyline and orphenadrine, highlighting the importance of metabolically generated nitroso intermediates in the mechanism-based inactivation of CYP450 enzymes (Sharma et al., 1996). Easily oxidizable C-H bonds and structural motifs such as methylenedioxyphenyl groups, tertiary or secondary amines, and heteroatom-rich aromatic systems are common features of this mechanism, while the balance between *K*_i_ and *k*_inact_ determines the overall potency of enzyme inactivation. However, due to the multiple variables in the assay protocol, enzyme isoforms, and conditions, the *k*_inact_, *K*_I,_ and *K*_i_ values should be compared and interpreted with caution.

In all the above cases, the CYP450 directly generates the inhibitory species responsible for its own inactivation, a hallmark of mechanism-based inhibition often also called “suicide inhibition”; however, we prefer not to use this terminology. The resulting metabolite-intermediate complexes block substrate access and oxygen activation without permanently altering the protein structure, explaining these inhibitors exhibit prolonged inhibition despite the absence of true covalent enzyme modification. Because these MBI inhibitors can produce clinically significant and long-lasting CYP450 inhibition, they present strong potential for drug-drug interactions and remain a major focus of drug metabolism and safety studies.

#### 3.2.2 Irreversible inhibitors

Irreversible inhibition, another class of mechanism-based or time-dependent inhibition, represents a CYP450 inhibition in which the enzyme either catalyses the bioactivation of the inhibitor to reactive metabolites or directly binds covalently to the electrophilic molecule. These intermediates/molecules irreversibly inactivate CYP450 by covalently modifying nucleophilic amino acid residues within the apoprotein, thereby disrupting the 3D structure and overall protein fold necessary for substrate recognition, electron transfer, oxygen activation, and substrate metabolism (Guengerich, 2018; Deodhar et al., 2020). The chemical nature of the reactive intermediate depends on the inhibitor scaffold. For example, Secobarbital and Gestodene contain olefinic (C=C) groups that undergo CYP450-mediated epoxidation to form electrophilic epoxide intermediates that bind covalently and are responsible for enzyme modification. In contrast, chloramphenicol is oxidised by CYP2C19 at its dichloroethyl moiety to form a gem-halohydrin intermediate, which subsequently rearranges to a highly reactive acyl chloride that covalently modifies lysine residues within the enzyme (see (Orr et al., 2012)). Thus, chloramphenicol is an irreversible inhibitor of CYP2C19, whereas it is a reversible (competitive) inhibitor of CYP3A4 and CYP2D6 (Park et al., 2003). In addition to metabolite-mediated inactivation, reactive oxygen species (ROS), including superoxide and hydrogen peroxide often formed during metabolic reactions, can also contribute to irreversible CYP450 inactivation (Correia and Hollenberg, 2015; Guengerich, 2022).

Other examples include, furafylline which exhibits the high inactivation rate, *k*_inact_ = 0.87 min^-1^; however, its higher *K*_I_ of 23 µM limits its overall inhibitory potential (Kunze and Trager, 1993; Liu et al., 2013). Since this natural product has a similar K_i_ value, the reversible binding component is comparable to the covalent binding giving a partitioning ratio zero. Similarly, Clopidogrel has equal contribution from reversible and irreversible components to its binding affinity. Bergamottin (Lin et al., 2005), a furanocoumarin present in grapefruit, displays moderate inactivation of CYP3A4 *K*_I_ = 22.4 µM, and shows tighter reversible binding (K_i_ = 2.9 µM), and *k*_inact_ = 0.087 min^-1^. These values point towards a moderately faster irreversible binding. Thus, this drug undergoes oxidative activation of its furan ring, generating reactive intermediates responsible for the well-known grapefruit juice-drug interactions. Most of the drugs and drug-like compounds in this category (irreversible) have higher affinity for the respective CYP450 isoform and are expected to show an initial reversible inhibitor feature; however, this reversible component is often not observed because of higher inactivation rate constant (*k*_inact_) and/or differences in assay protocols which makes direct comparisons difficult.

Another example is Binimetinib (see Table 4; Figure 8), which is a potent CYP1A2 inhibitor that acts through both reversible and time-dependent mechanisms. Binimetinib (Dong et al., 2021) exhibits relatively strong reversible binding ( $K_{i}=2.8$ µM) and a weaker affinity for inactivation ( $K_{I}=15.47$ µM), giving partitioning ratio 4.53. This suggests that once bound, the inhibitor remains associated with CYP1A2 for a prolonged period (in agreement with the experiment), allowing oxidation and eventual inactivation despite its relatively slow $k_{\text{inact}}$ .

Mibefradil (Foti et al., 2011; Orr et al., 2012), on the other hand, displays a balanced dual-inhibition profile. Its low $K_{i}$ (1 µM) indicates strong reversible binding, while the comparable $K_{I}$ (2.3 µM) and higher $k_{\text{inact}}$ (0.40 min^-1^) indicate that the bound complex is rapidly converted into an inactive enzyme. In kinetic terms, Mibefradil rapidly transitions from a reversible inhibitor into an irreversible inactivator, thereby explaining its liability to cause drug–drug interactions. This is reflected in its partitioning ratio, which is closer to one.

Zileuton (Lu et al., 2003) has relatively weaker reversible binding affinity ( $K_{i}=50$ µM) and also a weaker time-dependent inhibition $K_{I}$ (117 µM), indicating that higher concentrations are required to occupy the enzyme and initiate inactivation. In addition, a moderate $k_{inact}$ (0.035 min^-1^) suggests slow conversion to the inactive complex. Consequently, Zileuton functions predominantly as a weak reversible inhibitor with slower irreversible binding. Additionally, these different types of inhibition can also be investigated using spectroscopic features before undertaking detailed kinetic studies. Thus, the characteristic spectral features and corresponding insights on mechanisms of inhibition are discussed in the next section.

## 4 Spectral assessment and classification of CYP450 inhibitors and interactions

The characteristic absorbance spectrum of CYP450 originates from the electronic transitions of its prosthetic heme group, specifically the iron-protoporphyrin IX complex coordinated by a conserved cysteine thiolate ligand. In the resting state, ferric CYP450 Fe (III) predominantly exists as a water-bound hexa-coordinate, low-spin complex. This low-spin configuration produces a Soret absorption maximum at approximately 417–420 nm (see Table 5; Figure 9) arising from π → π* electronic transitions. The oxidation state, spin state, and coordination environment of the heme iron strongly influence the transition within the porphyrin ring. Upon reduction of iron from the ferric to the ferrous state, the heme iron shows higher binding affinity to carbon monoxide. This ligand binding is usually studied using difference spectroscopy, in which the Soret band shifts to 450 nm (Frydendall and Scott, 2024), giving rise to the characteristic CYP450 spectrum from which the enzyme family derives its name.

**TABLE 5:** Characteristic spectral peaks and changes in spin of the CYP450 resting state induced by ligand binding. The spin state is represented by the multiplicity (doublet or sextet) given by M = 2 S + 1, where S is the total spin, S = Σ s, and s is the individual unpaired electron spin = 1/2.

Spectrum type	λ_max_ (positive)	λ_min_ (negative)	Interpretation/Mechanism	Spin state	Representative examples
Ferric CYP (absolute absorption)	417–418 nm (Soret)	---	Resting ferric CYP450 with water coordinated to the heme iron; low-spin Fe (III) state	Low spin (doublet spin state; S = 1/2)	All CYP450 isoforms
Reduced and CO-bound difference spectrum	450 nm (P450) 420 nm (P420) *	∼420 nm	CO-bound reduced enzyme; 450 nm peak indicates intact functional CYP450, whereas 420 nm indicates denatured or inactive CYP450 (P420)	---	All CYP450 isoforms
Type I difference spectrum	388–392 nm	417–420 nm	Substrate binding displaces the axial water ligand, converts the ferric CYP450 low-spin to high-spin state	High spin (sextet spin state S = 5/2)	Testosterone, coumarin, benzphetamine, and many CYP450 substrates
Type II difference spectrum	428–432 nm	390–395 nm	Direct coordination of ligand nitrogen (e.g., imidazole, triazole, pyridine) to ferric heme iron	Low spin (doublet spin state; S = 1/2)	Ketoconazole, fluconazole, voriconazole, and quinine

**FIGURE 9:**
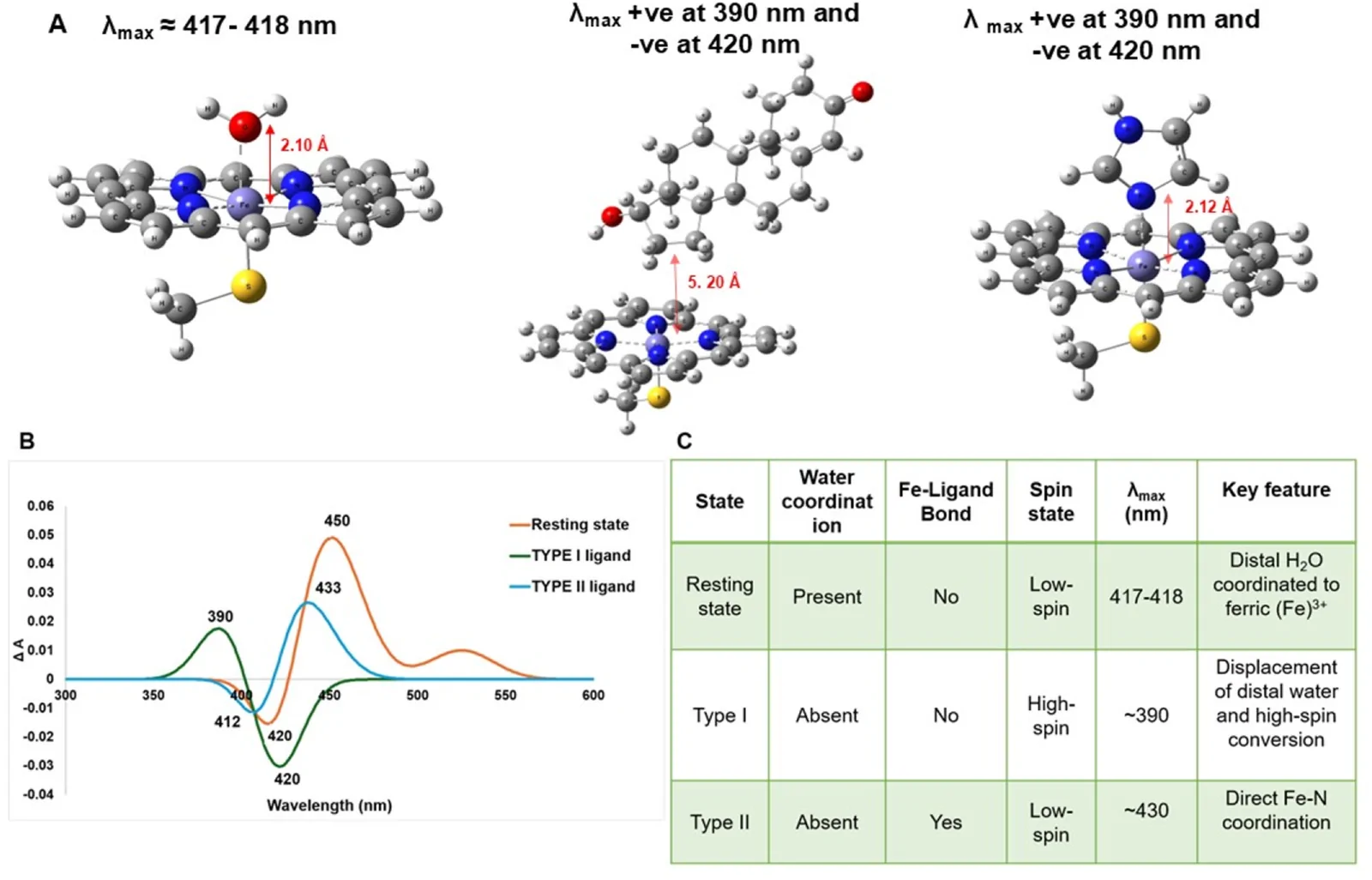
Structural and spectroscopic characteristics of various CYP450 ligand types. **(A)** Simplified heme-thiolate models representing the resting ferric state, Type I ligand binding, and Type II ligand binding, showing the corresponding coordination. **(B)** Representative difference spectra illustrating the characteristic spectral features associated with the resting, Type I, and Type II ligand binding. These spectra were manually plotted in excel using equation ΔAbs = $\sum_{i=1}^{3}A*\mathrm{EXP}\backslash (-\left({\left(\lambda -\lambda_{i}\right)}^{2}/\left(2*\sigma^{2}\right)\right)$ which is the sum of three Gaussian components centred at λ_i_ = 418, 535, and 570 nm, with amplitudes **(A)** = 0.05, −0.025, and 0.01 and standard deviations (σ) = 13, 14, and 15 nm, respectively. **(C)** Summary of the various features of resting state, and ligand types, including presence of water, ligand binding to Fe, spin state, characteristic λ_max_ values, and key features of each CYP450 binding mode.

The 450 nm absorbance results from the unique electron-donating properties of the proximal cysteine thiolate ligand, which stabilizes the Fe(II)-CO, complex and is useful for distinguishing CYP450 from other heme proteins. Binding of ligands to the distal side of the heme perturbs the coordination geometry and spin state of the iron, producing characteristic difference spectra that form the basis for classifying CYP450 inhibitors as Type I or Type II (Kumaki et al., 1978).

### 4.1 Type I binding spectral characteristics of CYP450 ligands

Type I inhibitors are generally substrate-like molecules that bind within the hydrophobic active site of CYP450 enzymes without directly coordinating to the heme iron. In the resting state of CYP450, the ferric heme iron Fe(III) exists predominantly in a low-spin hexacoordinate configuration, where the sixth coordination position is occupied by a water molecule. Upon binding of a Type I ligand, this coordinated water molecule is displaced from the active site, leading to a reorganisation of the heme environment and conversion of the Heme-Fe from a low-spin to a high-spin state. This spin-state transition alters the electronic distribution around the heme prosthetic group, thereby increasing the enzyme’s redox potential and facilitating electron transfer from the reductase partner (Sligar, 1976). Spectroscopically, this change is observed as a characteristic Type I difference spectrum (see Table 5; Figure 9), with an increase in absorbance near 385–390 nm and a corresponding decrease near 418–425 nm (Kumaki et al., 1978). The magnitude of the Type I spectral shift is influenced by the ability of the ligand to penetrate the active site and displace the axial water ligand. Unlike Type II inhibitors, which coordinate directly to the heme iron via a heteroatom, such as nitrogen, and stabilise the low-spin state, Type I ligands interact primarily through hydrophobic/electrostatic contacts with heme-Fe and amino acid residues lining the substrate-binding cavity. These interactions position the ligand near the catalytic centre while maintaining the heme iron in a catalytically competent high-spin configuration. Therefore, many Type I ligands are substrates or substrate-like competitive inhibitors rather than classical heme-coordinating inhibitors.

Structural, spectroscopic, and mechanistic studies have demonstrated that the low-spin-to-high-spin transition induced by Type I binding is a critical early event in the CYP450 catalytic cycle. The increase in the high-spin iron population enhances the reduction of Fe(III) to Fe(II), thereby promoting oxygen binding and the subsequent formation of reactive oxidising intermediates, such as Compound I. The protein active site residues and interactions also play a critical role in these binding and spin-state transition events, since, despite overlap in substrate scopes, many CYP450 isoforms retain substrate and inhibitor selectivity (Hirano et al., 2025). The study of classical Type I substrate ligands, including testosterone, midazolam, nifedipine, benzphetamine, aminopyrine, ethylmorphine, and lidocaine, has played a fundamental role in elucidating CYP450 substrate recognition and catalytic mechanisms.

Despite their structural diversity, these compounds share several common pharmacophoric characteristics, including hydrophobic aromatic or aliphatic frameworks, moderate to high lipophilicity, conformational flexibility, and the absence of strongly heme-coordinating heteroatoms. These properties promote binding within the hydrophobic substrate-binding cavity of CYP450 respective isoforms and avoid direct coordination to the heme iron. Testosterone, a prototypical CYP3A4 substrate, exemplifies the importance of a large hydrophobic steroidal scaffold in promoting strong active-site interactions and efficient spin-state conversion. Similarly, midazolam and nifedipine possess aromatic lipophilic frameworks and sufficient conformational flexibility to occupy the large CYP3A4 substrate-binding cavity, allowing productive positioning of oxidation sites near the heme prosthetic group. Benzphetamine and aminopyrine, which were among the earliest compounds used in CYP450 spectroscopy studies, contain hydrophobic aromatic regions and tertiary amine functionalities that facilitate active-site binding while maintaining a substrate-like orientation. Ethylmorphine employs its rigid morphinan scaffold to position the ethoxy group near the catalytic centre for O-deethylation, whereas lidocaine combines an aromatic ring and amide linker, and tertiary amine to establish favourable hydrophobic interactions within CYP3A4 and CYP1A2 active sites.

Collectively, these compounds demonstrate that Type I binding is primarily governed by hydrophobic interactions, molecular shape, lipophilicity, and the presence of accessible metabolic soft spots, rather than by direct heme coordination. Consequently, Type I ligands are typically substrates (that induce low-spin to high-spin transitions) or substrate-like competitive inhibitors (that do not induce spin state transitions), providing important mechanistic insight into CYP450 substrate recognition, catalytic efficiency, and drug-enzyme interactions.

### 4.2 Type II binding spectral characteristics of CYP450 ligands

Type II inhibitors are among the most extensively studied classes of quasi-reversible CYP450 inhibitors and are typically represented by nitrogen-containing heterocyclic compounds, including imidazole, triazole, pyridine, and quinoline derivatives. Unlike Type I ligands, which primarily interact with the hydrophobic substrate-binding cavity, Type II inhibitors directly coordinate a heteroatom, most commonly nitrogen, to the ferric heme iron [Fe(III)] located within the porphyrin ring of the CYP450 active site. This interaction stabilises the heme iron low-spin state and produces a characteristic red shift in the Soret band of the difference spectrum, with a maximum and minimum typically observed between 425–435 nm and 390–410 nm, respectively. The appearance of this spectral signature has therefore become a hallmark for identifying direct heme-coordinating inhibitors.

The inhibitory potency of Type II ligands is not solely determined by their ability to coordinate the heme iron. High-affinity inhibition generally results from a combination of strong iron-nitrogen coordination and favourable interactions within the hydrophobic substrate-binding site. Consequently, molecular properties such as lipophilicity, basicity, steric hindrance, conformational flexibility, and binding orientation significantly influence inhibitor potency and selectivity. Compounds capable of simultaneously engaging the heme iron and hydrophobic recognition regions of the active site typically exhibit stronger inhibition than molecules that rely exclusively on heme coordination (Correia and Hollenberg, 2015). Several clinically relevant drugs illustrate these structure-activity relationships, such as abiraterone, letrozole, anastrozole, and ketoconazole (see Figure 2). In abiraterone, pyridine nitrogen directly coordinates the CYP17A1 heme iron, while the steroidal scaffold occupies the hydrophobic active-site cavity. This interaction produces a Type II spectral shift with an absorbance -ve at 402 nm and +ve at 424 nm (Devore and Scott, 2012). In letrozole, the pyridine-like nitrogen of the triazole ring coordinates the CYP19A1 heme iron and the letrozole–aromatase complex showed a Type II spectrum with a crest at 422 nm and a trough at 394 nm (Hong et al., 2007). Two cyanophenyl groups of letrozole provide additional hydrophobic interactions that stabilise the inhibitor within the active site. Similarly, the imidazole nitrogen of ketoconazole coordinates the CYP3A4 heme iron, producing a characteristic Type II spectral response characterised by a trough near 390 nm accompanied by a crest around 425 nm (Peter Guengerich et al., 2020).

In comparison to ketoconazole, cimetidine, which also contains an imidazole ring, coordinates directly to the ferric heme iron and generates a characteristic Type II spectrum. However, despite sharing a similar heme-binding motif, ketoconazole is substantially more potent than cimetidine as a CYP450 inhibitor. This difference is largely attributed to ketoconazole’s greater lipophilicity (logP = 3.7) and larger molecular size, which enables extensive interactions within the hydrophobic active-site cavity. Crystallographic and molecular modelling studies have shown that ketoconazole adopts an extended conformation within CYP3A4, enabling simultaneous interaction with the heme prosthetic group and multiple hydrophobic recognition sites distributed throughout the active-site cavity. In contrast, cimetidine’s relatively low lipophilicity (logP = 0.4) limits its ability to establish stabilising hydrophobic contacts, resulting in weaker inhibition (see Section 6.1 for affinity data). A similar trend is observed with fluconazole, which contains a triazole ring that effectively coordinates to the heme iron but exhibits weaker CYP450 inhibition than ketoconazole due to its reduced hydrophobicity and lower affinity for the reversible binding in the substrate-binding cavity. Pyridine-containing compounds such as metyrapone and indinavir also inhibit CYP450 enzymes through direct heme coordination, while quinoline derivatives, including quinidine, quinine, and ellipticine, represent another important class of Type II ligands. Collectively, these examples demonstrate that optimal CYP450 inhibition requires a balance between heme coordination and complementary interactions within the active site. The imidazole ring functions as the primary heme-binding moiety, whereas the dichlorophenol and dioxolane-containing aromatic regions occupy the large hydrophobic substrate-binding cavity.

Several residues within the CYP3A4 substrate-recognition sites contribute to ketoconazole binding, including Phe57, Phe108, Ile120, Phe213, Phe215, Phe241, Ile301, Phe304, Ala305, Thr309, and Leu482. Among these residues, Phe304, Ala305, and Thr309 are positioned near the heme prosthetic group and play important roles in orienting the imidazole ring toward the ferric heme iron, thereby facilitating optimal iron–nitrogen coordination. Additional hydrophobic interactions involving aromatic and aliphatic residues stabilise the inhibitor within the active-site cavity and contribute to its high affinity and broad inhibitory profile against multiple CYP450 isoforms. Although ketoconazole is traditionally classified as a reversible Type II inhibitor based on its spectroscopic behaviour, kinetic studies have demonstrated that its inhibitory mechanism is more complex. CYP3A4 possesses a large and conformationally flexible active site capable of accommodating multiple ligands simultaneously. Consequently, ketoconazole can bind not only to the free enzyme but also to enzyme–substrate complexes, giving rise to mixed inhibition kinetics characterised by increases in the apparent K_m_ and decreases in V_max_. This behaviour reflects the cooperative and allosteric properties of CYP3A4 and highlights the distinction between spectroscopic classification and kinetic mechanism. Thus, ketoconazole represents a classic example of a compound that exhibits Type II spectral binding while simultaneously displaying mixed inhibition kinetics.

Importantly, CYP450 inhibition mechanisms are not always mutually exclusive, and a single molecule may exhibit multiple inhibitory mechanisms depending on its structural features. A notable example is PH-302, a potent and selective inducible nitric oxide synthase inhibitor that contains both an imidazole ring and a methylenedioxyphenyl moiety. In this case, the imidazole group mediates reversible Type II inhibition through direct heme coordination, whereas metabolic activation of the methylenedioxyphenyl functionality generates a metabolite–intermediate complex that produces time-dependent pseudo-irreversible inhibition. This dual mechanism illustrates the complexity of CYP450 inhibition and demonstrates that spectroscopic classification alone may be insufficient to fully describe inhibitor behaviour. Comprehensive mechanistic investigations incorporating spectroscopic, kinetic, structural, and metabolic studies are therefore essential for accurately characterising CYP450 inhibitors and predicting their potential to cause clinically significant drug–drug interactions (Kamel and Harriman, 2013).

## 5 FDA guidelines and decision tree for evaluating DDI potential of CYP450 substrates, inhibitors and inducers

A drug discovery company or group may identify the lead molecule using a combination of different screening approaches. These commonly involve a combination of molecular modelling, QSAR, drug design, high-throughput screening (HTS), and *in vitro* assays to evaluate efficacy and safety parameters. These methods are employed in early large-scale library screenings, from which medicinal chemists often determine the preferred scaffold(s) for developing SAR and advancing lead optimization. Additional ADMET prediction models are also often used to support risk assessments and finalize scaffolds and SAR studies. Despite widespread use in early discovery, the actual strategy for screening scaffolds and identifying lead molecules varies widely across pharma companies and other research groups. Thus, FDA and other regulatory agencies have never provided detailed guidelines for lead-optimization strategies; rather, they focus on the final molecule and the essential data type and quality required to propose a new chemical entity for clinical investigations.

Pharmaceutical Research and Manufacturers of America (PhRMA) and the US-FDA (Bjornsson et al., 2003; Grimm et al., 2009; U.S. Food and Drug Administration, 2020) have provided industry guidelines for investigating *in-vitro* drug interaction studies Cytochrome P450 enzyme and transporter mediated drug interactions (U.S. Food and Drug Administration, 2020). These guidelines primarily focus on the potential for DDIs arising from Phase I and II enzyme-mediated metabolism, as well as the potential for induction and inhibition of these metabolic processes. In this review, our focus is on interactions with CYP450 as a potential substrate and inhibitor, and the interested readers are referred to the above-mentioned guidance documents for details of induction studies (although a brief description is provided below). The workflow (see Figure 10A) begins by determining whether the lead compound is a CYP450 substrate. Reaction phenotyping studies can be performed using either 1) rCYP450 panel studies, which utilize reduction in metabolism of probe substrates to estimate inhibition, 2) selective chemical inhibition of CYP450 isoforms in hepatocyte cultures, or 3) a combination of hepatocyte incubation followed by rCYP450 studies. The rCYP450 panel studies are performed by incubating the compound with individual recombinant CYP450 isoforms, including CYP1A2, CYP2B6, CYP2C8, CYP2C9, CYP2C19, CYP2D6, and CYP3A4, in the presence of the reductase-NADPH system. Metabolite formation rates are quantified using LC–MS/MS, and the fraction metabolized (*f*_*m*_) by each CYP450 isoform is calculated.

**FIGURE 10:**
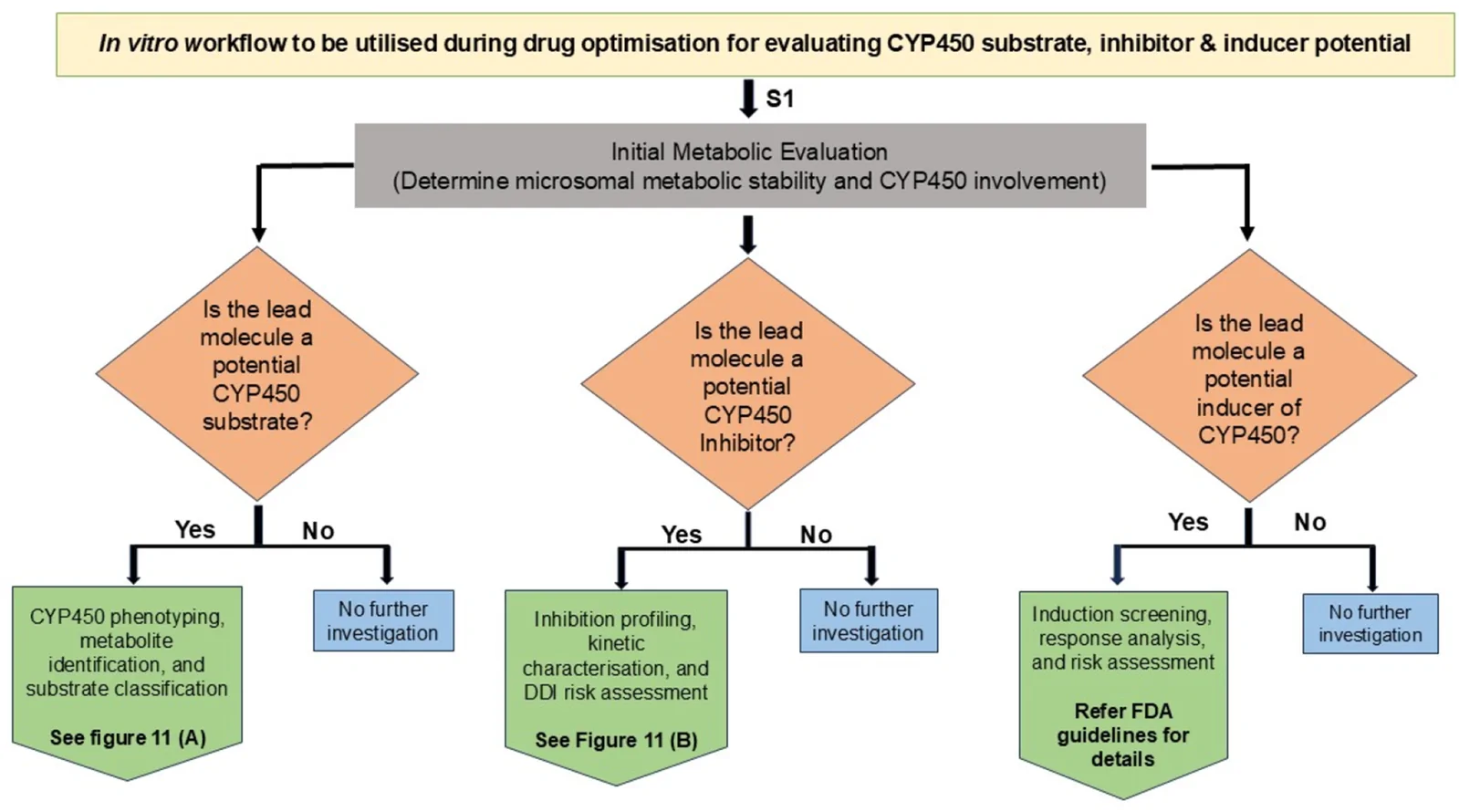
*In vitro* workflow for evaluating CYP450 substrate, inhibitor, and inducer potential. Following the initial step 1 (S1) of metabolic evaluation, compounds are taken up for substrate, inhibitor, or inducer assessment. Substrate and inhibitor pathways are further evaluated using the workflows shown in Figures 11A,B, respectively, while compounds identified as potential inducers undergo induction screening and risk assessment (Bjornsson et al., 2003; Grimm et al., 2009; U.S. Food and Drug Administration, 2020).

### 5.1 Estimating the CYP450 isoforms substrate potential (for lead compounds)

If a single CYP450 enzyme accounts for >25% of metabolism (*f*_m_), the compound is classified as a sensitive substrate. Such compounds have potential for CYP450-mediated DDIs, and additional PBPK modelling or clinical DDI evaluations are essential. For a substrate undergoing metabolism by multiple enzymes, the overall metabolic rate can be described as the sum of the individual Michaelis–Menten kinetics. Assuming classical Michaelis–Menten behaviour, the total metabolic rate ( $v_{\text{total}}$ ) is expressed as;

Michaelis-Menten behaviour for the total metabolic rate of a substrate metabolised by multiple enzymes (Equation 5).

$$v_{total}=\sum_{i=1}^{n}\frac{V_{\max,i\;}\left[S\right]}{K_{m,i}+\left[S\right]}$$ (5)

where $v_{\text{total}}$ is the total rate of metabolism, $V_{\max,i}$ is the maximum catalytic rate of the enzyme i , $\left[S\right]$ is the substrate concentration, and $K_{m,i}$ is the Michaelis-Menten constant of the enzyme i (Lee et al., 2024). The fractional contribution of an individual enzyme to the overall metabolism can be estimated from its reaction velocity relative to the total metabolic rate. However, for many potent drugs, therapeutic concentrations are typically much lower than the enzyme $K_{m}$ ( $\left[S\right]\ll K_{m}$ ). Under these low-substrate conditions, the Michaelis–Menten equation simplifies to: $v_{i}\approx \frac{V_{\max,i}}{K_{m,i}}\left[S\right]$ where the term $\frac{V_{\max,i}}{K_{m,i}}=CL_{\mathrm{int},i}$ (intrinsic clearance due to the enzyme i . Accordingly, the metabolic rate for each enzyme can be expressed as $v_{i}\approx CL_{\mathrm{int},i}\left[S\right]$ , and the total metabolic rate becomes:

Relationship between total metabolic rate and the intrinsic clearance (Equation 6)

$$v_{total}\approx \left[S\right]\sum_{i=1}^{n}{CL}_{\mathrm{int},i}$$ (6)

Thus, at typical therapeutic concentrations (sub-micromolar), the contribution of each enzyme to overall metabolism is determined by its intrinsic clearance relative to the sum of the intrinsic clearances of all contributing enzymes. The fraction metabolised ( $f_{m}$ ) by enzyme i can therefore be estimated, and the percentage contribution of the enzyme is be given by

Calculation for the percentage metabolic contribution of the enzyme in drug metabolism (Equation 7)

$$j=100\times \frac{\left(\frac{V_{\max,j}}{K_{m,j}}\right)}{\sum_{i=1}^{n}\left(\frac{V_{\max,i}}{K_{m,i}}\right)}$$ (7)

However, if the compound does not show significant CYP450-dependent metabolism, it can be declared a CYP450 non-substrate, and the workflow can logically progress to evaluation of CYP450 inhibition potential (See Figure 10).

### 5.2 Estimating the CYP450 inhibitory potential (of lead compounds)

As per US-FDA guidelines, separate studies are required to confirm reversible, time-dependent inhibitory potential of investigational molecules. An initial screen to detect significant inhibition is followed by separate mechanistic assays for reversible and irreversible inhibition. This leads to additional experimentation involving repeated *IC*_50_ (*K*_i_) measurements (see Table 6). A step-by-step comparison of the two protocols reveals that 4 out of 11 steps are identical in Table 6 and a modified workflow discussed below (see Figures 10, 11) can potentially save resources, effort, and time while providing the same evaluation. The time-dependent inhibition (TDI) protocol differs primarily by including an additional pre-incubation step followed by IC_50_ shift analysis to confirm the presence or absence of TDI. Based on this observation, the modified workflow provides a framework for assessing both reversible and irreversible inhibition within a single protocol.

**TABLE 6:** A comparison of US-FDA recommended stepwise separate protocols for reversible vs. irreversible CYP450 inhibitors (U.S. Food and Drug Administration, 2020).

Step #	Protocol step	Reversible inhibitor	Time dependent inhibitor
S1	HLM or rCYP450 + inhibitor + probe substrate	YES	YES
S2	Preincubation period	30 min	30 min
S3	IC_50_ determination	YES	YES
S4	TDI screening:30 min pre-incubation with NADPH	YES	NA
S5	IC_50_ shift calculation (>1.5 fold)	NA	Inhibition progressively increases with incubation time
S6A	Reversible inhibition: Kinetic characterisation ( $K_{i}$ , IC_50_)	YES	NA
S6B	Time-dependent inhibition: *K*_I_ and *k*_inact_ determination	NA	Characterised by *K*_I_ and *k*_inact_
S7	Protein dialysis	NA	Enzyme activity not fully restored; recovery may require new enzyme synthesis (irreversible inhibitor)
S8	Ferricyanide recovery assay	NA	YES
S9	Quasi-irreversible vs. standard irreversible inhibitor classification	NA	YES
S10	R_1_-based DDI risk assessment	YES	NA
S11	Calculate *k*_obs_ *and* R_2_ R_2_-based DDI risk assessment	NA	YES

**FIGURE 11:**
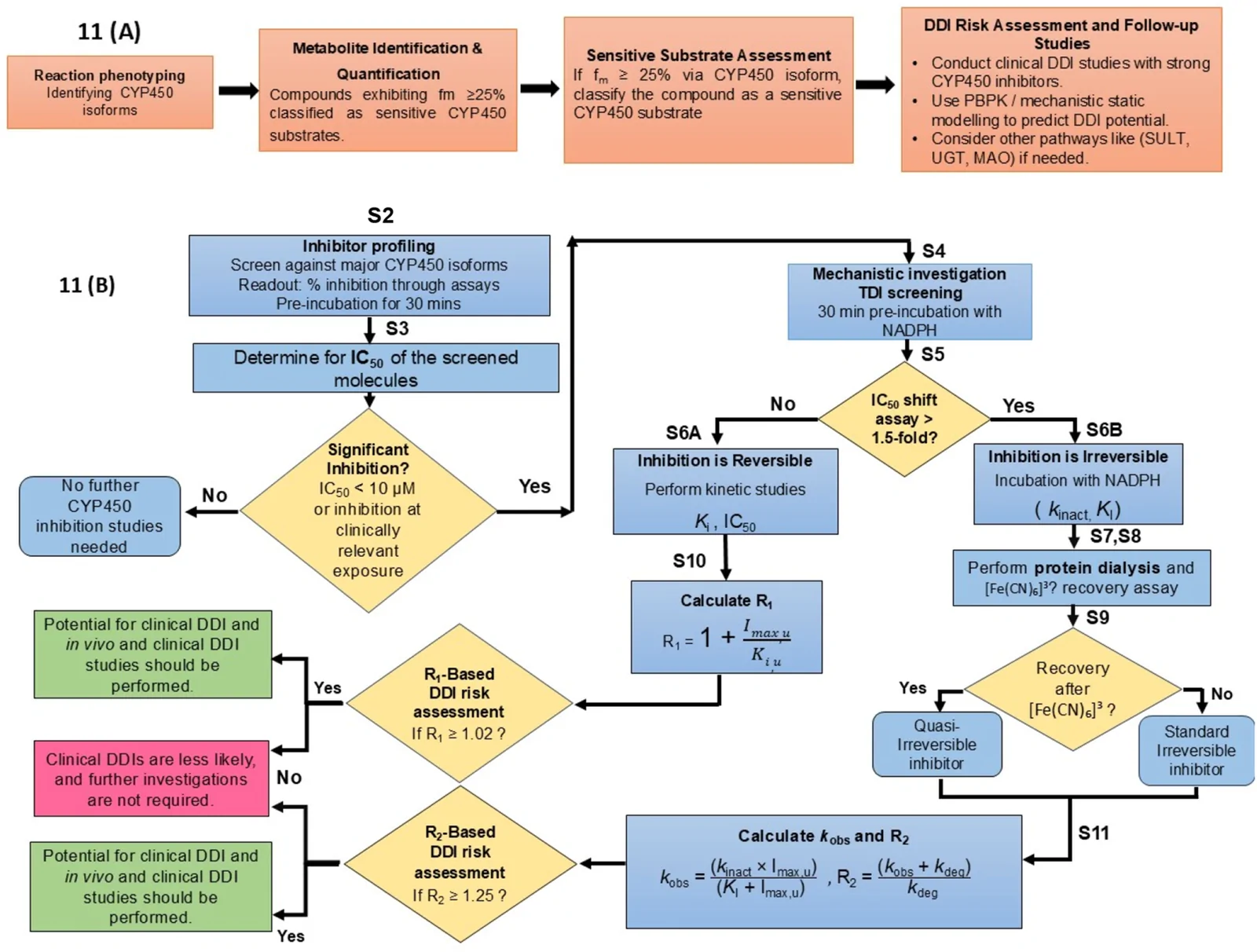
Detailed *in vitro* workflows for CYP450 substrate and inhibitor evaluation. **(A)** Workflow for CYP450 substrate evaluation, including reaction phenotyping, metabolite identification and quantification, sensitive-substrate assessment, and subsequent DDI risk assessment. **(B)** Workflow for CYP450 inhibitor evaluation, including initial screening and $IC_{50}$ determination, followed by mechanistic assessment of reversible or time-dependent inhibition. Reversible inhibitors are characterised using *K*_i_ and $IC_{50}$ , whereas irreversible inhibitors are evaluated using *k*_inact_ and *K*_I_, followed by protein dialysis and ferricyanide recovery assays. DDI risk is subsequently assessed using R_1_ and R_2_ calculation-based approaches. Here, *I*_max,u_ denotes the maximum unbound plasma inhibitor concentration, *K*_i,u is_ the unbound inhibition constant, *k*_obs_ is the observed inactivation rate constant, and *k*_deg_ is the enzyme degradation rate constant. (Bjornsson et al., 2003; Grimm et al., 2009; U.S. Food and Drug Administration, 2020).

The proposed workflow is not conceptually different from the US FDA *in vitro* guidance for CYP450 substrate and inhibition studies; however, it integrates the key elements of both assays into a single workflow. This streamlined protocol can reduce resource requirements while enabling simultaneous evaluation of reversible and irreversible inhibition, providing a practical, efficient strategy to the research community. The test compounds are initially screened against major CYP450 isoforms using HLMs or rCYP450 enzymes, using CYP450-selective probe substrates. The compound is incubated at multiple concentrations with each probe substrate in the presence of NADPH, and metabolite formation is quantified by LC-MS/MS or fluorescence assays. The percentage inhibition relative to the vehicle control is calculated. Potential CYP450 inhibitors are initially identified through inhibition screening, typically at a single inhibitor concentration (10–30 μM), in which compounds exhibiting significant inhibition (≥50%) are taken forward for IC_50_ determination and additional preincubation steps to estimate IC_50_-shift.

This IC_50_-shift calculation is followed by determination of (K_I_) and (*k*_inact_), only when IC_50_-shift >1.5-fold. This represents the primary experimental strategy for differentiating reversible and time-dependent CYP450 inhibitors during drug discovery and drug–drug interaction assessment (Berry and Zhao, 2008; Perloff et al., 2009; Grimm et al., 2009). Reversible inhibitors generally exhibit little or no change in inhibitory potency following preincubation, resulting in an IC_50_ shift of less than 1.5-fold and negligible enzyme inactivation; thus K_I_ ≈ K_i_ and rate constant *k*_inact_ ≈ 0 is usually not measured. In contrast, time-dependent inhibitors show increased inhibition after preincubation, characterised by IC_50_ shifts of ≥1.5-2-fold, progressive loss of enzyme activity with increasing incubation time, and measurable K_I_ and *k*_inact_ values. For reversible inhibitors, enzyme kinetic experiments are performed across a range of inhibitor and probe-substrate concentrations to determine IC_50_, *K*_i_, *K*_m_, and V_max_ values. Furthermore, to address the potential for clinical drug-drug interactions due to reversible inhibition R_1_ values are estimated. This parameter R_1_ is defined as,

Equation for the R_1_ parameter calculation for reversible inhibitors (Equation 8).

$$R_{1}=1+\frac{I_{\max,u}}{K_{i,u}}$$ (8)

where: $I_{\max,u}$ is the maximal unbound plasma concentration of the interacting drug at steady state and $K_{i,u}$ is the unbound inhibition constant for the *i*th enzyme.

Here, “interacting drug” means the victim drug whose metabolism is affected by the inhibitor (perpetrator drug). For reversible inhibitors, if R_1_ < 1.02, no additional investigation is performed. This means that the ratio *I*_max_,_u_/*K*_i,u_ < 0.02, and the unbound inhibitor concentration in plasma should remain 50 times lower than the inhibition constant (*K*_i,u_). When R_1_ > 1.02, further mechanistic investigation or clinical DDI studies are essential. If the IC_50_-shift >1.5-fold, further TDI studies are essential (see Figures 10, 11) to estimate the potential of DDIs. In these, the compound is pre-incubated with HLMs and NADPH for different time periods before the addition of the probe substrate to allow enzyme inactivation. The inactivation parameters *k*_inact_ and *K*_I_ are experimentally determined. The observed inactivation constant (*k*_obs_) is calculated using:

Equation for calculating the observed inactivation constant (Equation 9),

$$k_{obs}=1+\frac{k_{i\text{nact}}*50*I_{\max,u}}{K_{I,u}+50*I_{\max,u}}$$ (9)

where: $k_{\text{inact}}$ is the maximal inactivation rate, $K_{I,u}$ is the unbound inhibitor concentration at half-maximal inactivation and $I_{\max,u}$ is the maximal unbound inhibitor concentration. The FDA then recommends calculation of the irreversible inhibition parameter (R_2_):

Equation for calculating the irreversible inhibition parameter R_2_ (Equation 10)

$$R_{2}=1+\frac{k_{obs\;}+k_{\deg}}{k_{\deg}}$$ (10)

where: $k_{\deg}$ is the natural degradation rate constant of the CYP50 enzyme and $k_{obs\;}$ is the observed enzyme inactivation rate. If R_2_ < 1.25, no further investigation is generally required; when R_2_ > 1.25, mechanistic modelling or clinical DDI studies are recommended.

In CYP450 induction studies, primary human hepatocytes from multiple donors are incubated with increasing concentrations of the investigational compound for 48–72 h. Known inducers such as rifampicin or omeprazole are used as positive controls. The induction of CYP1A2, CYP2B6, and CYP3A4 are evaluated by measuring both mRNA expression and enzyme activity. Quantitative RT-PCR is used to determine the fold induction of CYP450 mRNA, while CYP450 activity assays are performed using selective probe substrates. Concentration-response curves are generated to determine the Relative Induction Score (RIS) using maximum induction effect (E_max_) and EC_50_ parameters using the formula RIS = *E*_max *_ *I*_max,u_/*EC*_50_+ *I*_max,u_, where: E_max_ = maximum induction effect, EC_50_ = concentration producing half-maximal induction, I_max=_maximal unbound plasma concentration of interacting drug at steady state.

The FDA has recommended another approach using a basic induction model for calculating the R_3_ parameter:

$$R_{3}=\frac{1}{1+d*\left(\frac{E_{\max}\;*\;10\;*\;I_{\max,u}}{{EC}_{50}+10\;*\;I_{\max,u}}\right)}$$

where: d = scaling factor (usually 1), *E*_max_ = maximal induction effect, *EC*_50_ = concentration causing half-maximal induction, *I*_max,u_ = maximal unbound plasma concentration of the interacting drug. Compounds exhibiting $R_{3}\leq 0.8$ are considered potential CYP450 inducers and may require further mechanistic modelling or clinical DDI studies.

CYP450 inducers can reduce systemic exposure of co-administered drugs metabolized by the same CYP450 enzyme. Such compounds are recommended for further PBPK modelling and clinical DDIs studies. Overall, the workflow follows a logical strategy in which the investigational compound is evaluated for potential CYP450 metabolism and possible inhibition and induction of the CYP450 enzyme systems. This integrated approach enables comprehensive characterisation of CYP450-mediated metabolic pathways and drug-drug interaction (DDI) liabilities during early drug development.

## 6 Factors regulating therapeutic benefits (friend behaviour) or adverse effects (foe behaviour) of CYP450 inhibitors

Differentiating the potential of a therapeutically beneficial CYP450 inhibitors from those that cause clinically significant DDIs during drug discovery and development remains a major challenge. Conventional classification criteria used in *in-vitro* studies, including Type I/Type II spectral binding patterns and mechanisms of inhibition (reversible, irreversible, or mechanism-based), are not useful for prediction of clinical responses. Since an equally potent inhibition has the same effect on metabolic profile of a victim drug irrespective of the mechanism of inhibition (reversible or time-dependent). Thus, the therapeutic benefits or adverse effects observed in clinical trials or real world setting after administering a CYP450 inhibitor are governed by multiple pharmacodynamic, pharmacokinetic, disease specific, and patient centered factors. These factors generally include the following; 1) isoform selectivity estimated using pharmacodynamic parameters (affinity, and potency), 2) effect on pharmacokinetic parameters of CYP450 substrate drugs including metabolic clearance, 3) prodrug activation, 4) reactive metabolite formation, and 5) co-administered drugs. The isoform selectivity factor often presents as selective inhibition of disease-associated (e.g., CYP19A1 and CYP17A1) or drug-metabolizing CYP450 isoforms (e.g., CYP3A4 and CYP2D6). Since the inhibition of disease-associated CYP450s is usually highly selective, it often turns out to be beneficial since these CYP450 isoforms are pharmacological targets. Similarly, inhibition of drug-metabolizing CYP450 isoforms, which reduces the metabolic clearance of co-administered drugs, may allow dose reduction and serve as a pharmacokinetic booster (Krauß and Bracher, 2018). However, the same mechanism can also cause drug–drug interactions (Schroeter et al., 2025), via overactivation of targets (on-target) and off-target side effects for drugs that heavily depend on CYP450s for clearance. In contrast, inhibition of CYP450-mediated prodrug activation, essential for therapeutic response, decreases the formation of active metabolites and reduces efficacy. Whereas the inhibition of reactive metabolite formation caused by inhibition of drug-metabolizing CYP450s may prevent bioactivation-mediated toxicity. Based on the effect of these factors, the CYP450 inhibitors may be loosely classified as a friend or a foe. The detailed explanation of these factors and their effect is discussed below.

### 6.1 Isoform-selective CYP450 inhibitors that gives therapeutic benefit (A friend or foe)

Isoform selectivity is usually the most informative parameter for analysing multitarget activities and structure-promiscuity relationships (SPRs) (Dimova and Bajorath, 2018) and can also be useful for differentiating CYP450 inhibitors in the present context. The clinical utility of a CYP450 inhibitor depends not only on its potency toward the intended therapeutic isoform but also on its relative potency toward drug-metabolising isoforms. Compounds/drugs highly selective for the target usually have minimal potential to inhibit off-targets and cause drug–drug interactions (Dixit and Singh, 2021). This approach has been extensively used by medicinal chemists in lead optimisation; however, a direct assessment of selectivity in situations where both target and off-target are CYP450s has not been performed. Thus, we analysed the pairs of potency values for various drugs where both the target and off-target are CYP450s. Analysis of clinically used CYP450 inhibitors (see Table 7) suggests that a ΔpIC_50_ greater than 1 log unit (approximately ≥10-fold selectivity) is generally associated with a favourable therapeutic profile, whereas ΔpIC_50_ less than 1 indicates a higher likelihood of binding with drug-metabolising CYP450 (off-target) and an increased risk of clinically significant drug–drug interactions.

**TABLE 7:** Isoform selectivity (**ΔpIC**_**50**_) as a parameter for differentiating CYP450 inhibitors into friends or foes.

S. No.	CYP450 inhibitor	CYP450 target isoform	Target isoform potency (pIC_50_)	Drug-metabolizing CYP450 isoform	Drug-metabolizing CYP450 potency (pIC_50_)	ΔpIC_50_	Predicted friend or foe	Agreement with clinical experience
1	Osilodrostat	CYP11B1	8.54	CYP3A4	5.31	3.21	Friend	No
2	Abiraterone	CYP17A1	6.09	CYP2D6*	6.80	−0.71	Foe	Yes
3	Letrozole	CYP19A1	7.94	CYP2C19*	5.05	2.44	Friend	Yes
4	Ketoconazole	CYP51	6.73	CYP3A4	4.10	2.63	Friend	Yes
5	Voriconazole	CYP51	5.55	CYP3A4	4.88	0.66	Foe	Yes
6	Fluconazole	CYP51	5.25	CYP3A4	4.49	0.75	Foe	Yes
7	Itraconazole	CYP51	5.44	CYP3A4	6.04	−0.60	Foe	Yes

* Converted experimental K_I_, value into IC_50_ and pIC_50_ value.

Consistent with this criterion, letrozole, ΔpIC_50_ = 2.44, selectively inhibits CYP19A1 over CYP2C19 (see Table 7) and is also known to exhibit minimal clinical CYP450-mediated interactions (Muftuoglu and Mustata, 2010). Similarly, ketoconazole (Ekins et al., 2007) (ΔpIC_50_ = 2.63) preferentially targets fungal CYP51, despite significant potential to inhibit CYP3A4 at higher concentrations it remains clinically useful for treating topical fungal infections. In contrast, abiraterone (ΔpIC_50_ = −0.71) (Njar and Brodie, 2015), voriconazole (ΔpIC_50_ = 0.66), fluconazole (ΔpIC_50_ = 0.75) (Shrestha et al., 2017), and itraconazole (Ekins et al., 2007) (ΔpIC_50_ = −0.60) inhibit drug-metabolising CYP450 enzymes with potency comparable to or exceeding that of their therapeutic targets and exhibit significant clinical drug-drug interactions.

This prediction is consistent with clinical evidence showing that inhibition of CYP3A4 by azole antifungals, particularly itraconazole, markedly increases the exposure of co-administered CYP3A4 substrates and can lead to serious adverse events. Overall, the proposed selectivity criterion seems to be clinically useful for predicting the potential of CYP450 inhibitors to be useful or detrimental in combination therapies. The notable exception is osilodrostat (Grombein et al., 2015), which exhibits high *in vitro* selectivity for CYP11B1 over CYP3A4 (ΔpIC_50_ = 3.21) and would therefore be predicted as a “friend.” However, clinical studies have reported CYP3A4-mediated drug interactions and an increased risk of adverse reactions. This discrepancy indicates that *in vitro* selectivity alone may not be sufficient to predict clinical outcomes and additional factors, can be equally important in deciding the type of interactions and their consequences. Nevertheless, overall, this trend seems to support ΔpIC_50_ ≈ 1 log unit as a meaningful threshold for potentially distinguishing therapeutically selective CYP450 inhibitors from those likely to cause drug-drug interactions. Thus, drugs or clinical candidates with higher selectivity (ΔpIC_50_ > 1) are expected to be “Friends” and can be taken forward in drug development stages.

### 6.2 Effect of metabolism on drug action as a factor for differentiating friend vs. foe

Whether the effect on metabolism of a co-administered CYP450 inhibitor is therapeutically beneficial (Friend) or detrimental (Foe) to the main drug effect can be understood using five mechanistic factors: (i) whether the main drug requires CYP450-mediated bioactivation (PAMD), (ii) whether metabolism generates a toxic reactive metabolite (TRMF), (iii) the extent of CYP450-mediated metabolism, (iv) whether hepatic metabolism is the primary clearance pathway, and (v) the effect of metabolism on drug action (Table 8). Among these, the effect of metabolism on drug action appears to be the primary determinant of the interaction outcome. Analysis of the representative clinical examples suggests a consistent trend that when metabolism is Beneficial (B), because it activates a prodrug (e.g., ticagrelor, clopidogrel, codeine, and tamoxifen) or maintains appropriate systemic exposure through metabolic clearance, inhibition of the responsible CYP450 enzyme is more likely to produce an adverse drug interaction (Foe). Conversely, when metabolism is Detrimental (D) since it inactivates the parent drug, CYP450 inhibition tends to increase systemic exposure and enhance therapeutic efficacy or prolong drug action. This is consistent with the application of many CYP450 inhibitors as pharmacokinetic boosters (Friends, e.g., ritonavir and cobicistat) (Krauß and Bracher, 2018).

**TABLE 8:** Examples of CYP450 inhibitors that act as a friend or foe by inhibiting the metabolite conversion of the parent drug. Pro-drug activation required for the main drug’s action (PAMD), Beneficial (B), Detrimental (D), and Neutral (Neu). # Clinical experience of all the main drugs confirmed from US-FDA drug label.

S.N.	Main drug	Co-administer CYP450 inhibitor	PAMD	Metabolism’s effect on drug action	Predicted friend/Foe	Clinical experience^#^
1	Ticagrelor	Ketoconazole	Y	B	Foe	Foe
2	Clopidogrel	Omeprazole	Y	B	Foe	Foe
3	Codeine	Quinidine	Y	B	Foe	Foe
4	Tamoxifen	Letrozole	Y	B	Foe	Foe
5	Midazolam	Diltiazem	N	B	Foe	Foe
6	Diclofenac	Voriconazole	N	B	Foe	Foe
7	Venetoclax	Ritonavir	N	B	Foe	Foe
8	Bosutinib	Ketoconazole	N	B	Foe	Foe
9	Lapatinib	Ketoconazole	N	B	Foe	Foe
10	Darunavir	Ritonavir	N	D	Friend	Friend
11	Darunavir	Cobicistat	N	D	Friend	Friend
12	Atazanavir	Ritonavir	N	D	Friend	Friend
13	Ritonavir	Posaconazole	N	Neu	Foe	Foe
14	Lopinavir	Ritonavir	N	D	Friend	Friend
15	Acetaminophen	Fomepizole	N	D	Friend	Friend
16	Nirmatrelvir	Ritonavir	N	D	Friend	Friend

All the representative interactions were correctly classified based on the effect of the CYP450 inhibitor (Friend or Foe) on parent drug’s metabolism after including other factors like pro-drug activation. Whenever drugs require a CYP450-mediated bioactivation step for therapeutic effect, a CYP450 inhibitor will be a Foe (i.e., cause an adverse drug reaction). Examples include Ticagrelor and clopidogrel, where ketoconazole and omeprazole are contraindicated. In case of drugs that are extremely potent, need to be metabolized and excreted immediately after therapeutic action, inhibition of CYP450 leads to an increase in drug concentrations and adverse effects, i.e., the CYP450 inhibitor acts as a Foe. Representative Foe and drug combinations that increase drug concentration to toxic levels include Diltiazem-midazolam, Voriconazole-diclofenac, Ritonavir-venetoclax, Ketoconazole-bosutinib, and Ketoconazole-Lapatinib. It is worth noting the unique case of Ritonavir here, since it acts as a Friend while interacting with metabolism of some drugs (e.g., darunavir and Lopinavir), however, it becomes a Foe if co-administered with others, e.g., posaconazole and venetoclax. Thus, the behaviour of a perpetrator drug depends on the context of use which covers the factors mentioned above.

In contrast, ritonavir or cobicistat-mediated boosting of darunavir, atazanavir, lopinavir, and nirmatrelvir exemplify friendly interactions between drugs. This means that the therapeutic benefit of the drug is exclusive dependent on the CYP450 inhibition caused by the co-administered drug. Here the inhibition of hepatic metabolism keeps plasma concentrations of the active drug at therapeutic levels leading clinical effectiveness. Although derived from a representative dataset, these findings suggest that, alongside isoform selectivity, the effect of metabolism on drug action provides a practical mechanistic criterion for differentiating CYP450 inhibitors into “Friends” or “Foes”.

## 7 CYP450 inhibitor prediction using different computational models

Computational prediction of CYP450 inhibition has become an essential component of modern drug discovery since it is a major cause of DDIs, altered drug exposure, therapeutic failure, and adverse drug reactions. Consequently, predicting CYP450 inhibition is now routinely integrated into lead optimisation and ADMET assessment to reduce experimental cost, minimise late-stage attrition, and improve the safety profile of candidate drugs (Kato, 2020). A wide range of approaches have been developed in the last ∼40 years to model CYP450 reactions, substrates, inhibitors and interactions. This includes methods like QSAR, pharmacophore modelling, molecular docking, machine learning, and other hybrid methods. Several review articles have covered these methodologies, and discussed their predictive performance, strengths, and limitations (Verras et al., 2006; Sridhar et al., 2012; Dixit and Deshpande, 2016; Tyzack and Kirchmair, 2019; Kato, 2020).

In the above Section 6.1 isoform selectivity was identified as an important factor for differentiating therapeutic benefits from adverse effects (Friend vs. Foe behavior). The required selectivity, however, depends on the intended use of the CYP450 inhibitor. When inhibitors are targeted against non-drug metabolizing isoforms such as CYP17A1 and CYP19A1, high selectivity toward the therapeutic isoforms (vs. drug-metabolizing CYP450s) is desirable to minimize the potential for DDIs. In contrast, when CYP450 inhibitors are used to inhibit the metabolism of a co-administered drug, high selectivity toward the specific drug-metabolizing CYP450 isoform is required. Thus, meaning and benefit of selectivity depends on the context (Raut and Dixit, 2025).

An extensive literature search was performed to identify existing computational models that can predict CYP450 isoform selectivity and thus can potentially differentiate/predict the beneficial or detrimental effects of CYP450 inhibition. Several multitask, graph-based and multimodal approaches have been developed to predict CYP450 inhibition across major drug-metabolizing isoforms, including CYP1A2, CYP2C9, CYP2C19, CYP2D6, and CYP3A4. However, these models primarily classify molecules as inhibitor or non-inhibitor of individual CYP450 isoforms rather than providing a direct prediction of isoform selectivity. Selectivity also cannot be predicted simply by combining independently developed isoform-specific prediction models, because such a combination of two independent model is itself a new model which requires independent validation (which is currently not possible due lack of training data). Moreover, differences in other model parameters like dataset type, and size, applicability domains, and prediction error propagation would reduce the reliability of any combined selectivity model. Even after extensive literature search, we did not identify a model that can directly predict CYP450 isoform selectivity (Bender et al., 2026).

Thus, a review of the recent progress in the field of artificial intelligence (AI), deep learning, and large language models (LLMs), approaches for prediction CYP450 inhibitors is presented below. Considering the vast amount of literature on the topic (which has already been reviewed earlier (Verras et al., 2006; Sridhar et al., 2012; Dixit and Deshpande, 2016; Tyzack and Kirchmair, 2019; Kato, 2020) and recently (Plonka et al., 2021) we decided to focus on the newer models published in the last 5 years (Table 9).

**TABLE 9:** ML, deep learning, neural network and LLM-based CYP450 inhibition prediction models, developed in the last 5 years (2021–2025).

S.N.	Model	Dataset	Model approach and key features	Advantage	Limitation
1	CYPlebrity (2021)	PubChem, ChEMBL, and ADME datasets contain 18,815 molecules	Random forest with Morgan3 fingerprints	Reasonable performance even for compounds with limited structural similarity to the training data	SuperCYPsPred provided higher sensitivity while CYPlebrity showed comparable specificity
2	iCYP-MFE (2021)	PubChem BioAssay dataset contains total of 4,352	Multitask learning	Multitask learning improved the prediction performance compared with corresponding single-task models	Relatively less balanced datasets, possibility of residual errors
3	GCNN (2022)	PubChem database contained.14,683	Combining molecular graph and CYP450 binding-site residues	Improved molecular representation by with molecular graph features and CYP450 substrate-binding residues	Model remained limited by substantial class imbalance
4	DEEPCYPs (2023)	iCYP-MFE model dataset	FP + GNN multitask architecture	Model showed good performance with 0.90 AUC and 0.81 balanced accuracy	Predefined molecular fingerprints, does not explicitly incorporate CYP450 protein structural information
5	CYP3A4-TDI (2023)	PubChem databases contain 7914 unique including 581 TDI compound	Random under-sampling (RUS) and random over-sampling (ROS)	Dedicated TDI prediction	Model developed with imbalanced dataset contain limited experimentally confirmed TDI compounds
6	ML-augmented docking (2023)	∼50,000 curated compound for five major metabolising isoform	Docking + ligand features	Models gave more accurate prediction due to combining ligand and docking based features	Dataset did not provide sufficient chemical diversity
7	CYPi-DNN (2023)	PubChem BioAssay dataset containing 93,823 compounds	Deep neural network (DNN) based on MACCS fingerprints	Model achieved maximum AUC 0.97 for CYP1A2	Validation set chemical space was similar to the training dataset.
8	Permadi et al. (2025)	ChEMBL, PubChem, database contain 170,355 compounds	Multitask learning + missing-label imputation	Multitask learning and missing-label imputation may improve performance when experimental data are limited	Low external recall for CYP2B6. Showed applying this approach to chemically diverse dataset
9	Multimodal encoding network (MEN) (2025)	PubChem database contain ∼1,20000 molecules	Fingerprint + graph + protein sequence + MMA	Combined model achieved an ACC of 93.7% and AUC of 98.5% compared to individual encoders	Complex architecture, limited protein-side interpretability, reduce performance

The development of CYP450 inhibition models has progressively addressed limitations in data coverage, molecular representation, multitask learning, mechanistic information, and interpretability. To overcome this limitation the CYPlebrity (Plonka et al., 2021) model expanded the chemical space by integrating PubChem, ChEMBL, and ADME datasets that contained approximately 14,329–18,815 compounds. The Random Forest classifiers were used including Morgan3 fingerprints. The optimized models used 2048-bit fingerprints, 400 trees, a minimum split size of 16, and 16 features considered at each split. The models achieved MCC values of 0.62–0.70 and AUC values of 0.89–0.92 across the five CYP450 isoforms. Importantly, the models maintained reasonable performance even for compounds with limited structural similarity to the training data, with a proposed applicability threshold of approximately TC ≥ 0.5. Compared with SuperCYPsPred (Banerjee et al., 2020), CYPlebrity showed comparable overall performance, although SuperCYPsPred provided higher sensitivity while CYPlebrity showed comparable specificity and a higher average F-measure.

iCYP-MFE (Nguyen-Vo et al., 2022) a multitask deep-learning convolutional neural network (CNN) with molecular fingerprint-embedded framework for CYP1A2, CYP2C9, CYP2C19, CYP2D6, and CYP3A4 inhibitors prediction. The model was trained using experimentally validated PubChem BioAssay dataset contains total of 4,352 compounds for all five datasets. Multitask learning improved the prediction performance compared with corresponding single-task models, with AUC-ROC values of 0.91, 0.89, 0.86, 0.93, and 0.89 for CYP1A2, CYP2C9, CYP2C19, CYP2D6, and CYP3A4, respectively. Compared with SuperCYPs Pred and SwissADME (Daina et al., 2017), iCYP-MFE showed better performance for most isoforms. However, the study was limited by relatively less balanced datasets, incomplete statistical comparison with SwissADME, and the possibility of residual errors in the manually unverified large-scale curated datasets.

The unified graph convolutional neural network with an attention mechanism (GCNN) (Qiu et al., 2022) model was developed using PubChem database contained 14,683 unique compounds for five different isoforms. This model improved molecular representation by replacing predefined fingerprints with molecular graph features and incorporating CYP450 substrate-binding residues. Unlike fingerprint-based models, the proposed approach represented inhibitors as molecular graphs in which atoms and chemical bonds were encoded directly. A graph attention layer followed by graph convolution was used to learn ligand features, while each CYP450 isoform was represented by a 42-residue pseudo-sequence derived from key substrate-binding residues. This allowed the model to learn structural relationships within the molecule while also partially considering the CYP450 binding environment. This GCNN model achieved AUC values of 0.89–0.92 and MCC values of 0.58–0.68 across the five CYP450 isoforms on independent test datasets. Using the datasets used for developing iCYP-MFE, the model gave similar performance for four CYP450 isoforms, while iCYP-MFE showed better performance for CYP2D6. However, the model remained limited by substantial class imbalance, and the use of a fixed set of 42 CYP450 binding residues, which may not represent ligand-specific binding interactions.

DEEPCYPs (Ai et al., 2023) a multitask deep-learning platform used the previously prepared dataset of iCYP-MFE. This model integrated with predefined fingerprint features with learned graph representations. The model achieved average test-set values of 0.90 AUC and 0.81 balanced accuracy. It ranked first in AUC for CYP1A2, CYP2C9, CYP2C19, and CYP3A4, although iCYP-MFE performed better for CYP2D6. However, the model relies partly on predefined molecular fingerprints, it does not explicitly incorporate CYP450 protein structural information. Parallel studies discussed below expanded CYP450 prediction toward more mechanistically relevant endpoints and protein-level information.

(Xu et al., 2023) developed a CYP3A4-specific model for prediction of time-dependent inhibition (TDI) using preprocessed 7,914 unique compounds, including 581 TDI compounds from the PubChem database. To address the substantial class imbalance, random under-sampling (RUS) and random over-sampling (ROS) strategies were evaluated. The GCN–GraphConv model was trained on the RUS dataset, which provided the best performance for predicting inhibitors and achieved an AUC of 0.982 and balanced accuracy of 0.941 on the internal test set. The model was subsequently applied to a PAINS-filtered SPECS database containing 188,065 compounds, from which 15 compounds were selected for experimental validation. CYP3A4 TDI was experimentally assessed using NADPH-dependent IC_50_-shifts. The experimental validation achieved a balanced accuracy of 72%. Despite its strong internal performance, the model was developed using a highly imbalanced dataset with relatively few experimentally confirmed TDI compounds; thus, the wider applicability of this model needs to be tested further.

(Weiser et al., 2023) incorporated protein-ligand interaction information by combining ligand descriptors with docking-derived features. The study used curated CYP450 inhibition datasets for five major isoforms, containing approximately 10,000 compounds per isoform. Each compound was docked, and residue-level hydrogen-bond, van der Waals, and electrostatic interactions were extracted together with ligand descriptors such as molecular weight, logP, and Fsp3. Six machine-learning algorithms were evaluated, with gradient boosting and extreme gradient boosting (XGB) providing the best performance. Using XGB ensembles, the combined model achieved AUC values of 0.91, 0.85, 0.84, 0.88, and 0.88 for CYP1A2, CYP2C19, CYP2C9, CYP2D6, and CYP3A4, respectively. The authors also noted that the available dataset may not provide sufficient chemical diversity and that further high-quality experimental data are required for reliable extrapolation to new chemical space.

CYPi-DNN developed by (Chen et al., 2024) combined a large PubChem BioAssay dataset containing 93,823 compounds, with 57,380 compounds used for training and 36,443 for testing, with a fully connected deep neural network based on MACCS fingerprints. Molecular structures were represented using 166-bit MACCS/MCCS fingerprints and used as input to isoform-specific fully connected deep neural networks containing three hidden layers. Class weighting was applied to address the imbalance between inhibitor and noninhibitor classes, while dropout, batch normalisation, and L1/L2 regularisation were used to reduce overfitting. The models achieved AUC values of 0.97, 0.94, 0.94, 0.96, and 0.94 for CYP1A2, CYP2C9, CYP2C19, CYP2D6, and CYP3A4, respectively. Moreover, the validation chemical space was similar to the training space, limiting conclusions regarding performance on structurally novel compounds.

(Gong et al., 2024) systematically evaluated conventional machine-learning and deep-learning approaches for predicting inhibition of CYP2C9, CYP2D6, and CYP3A4 using unified datasets derived from PubChem BioAssays. The study compared multiple molecular representations, including fingerprints, physicochemical descriptors, molecular graphs, and SMILES sequences, with SVM, RF, LightGBM, XGBoost, CatBoost, GCN, GAT, GIN, Attentive FP, LSTM, CNN, and BERT algorithms. Among the conventional models, combined fingerprint/descriptor representations, particularly FCFP4 or ECFP4 with RDKit or Mold2 descriptors, coupled with XGBoost or CatBoost, consistently produced the best test-set performance, with AUC values of approximately 0.92. In contrast, graph- and SMILES-based deep-learning models generally achieved lower AUC values. External validation further supported the superiority of the conventional ML models, although the external datasets occupied chemical spaces like the training data.

More recent studies have focused on data scarcity and multimodal learning (Permadi et al., 2025). developed a multitask deep-learning model to address the limited availability of inhibition data for CYP2B6 and CYP2C8. Experimental IC_50_ data were collected from ChEMBL, PubChem, and previously published datasets. This gave 12,369 compounds covering seven CYP450 isoforms. A graph convolutional network was initially developed as a single-task model, but its performance was poor for CYP2B6 and CYP2C8, with F1 scores of 0.402 and 0.550 and Cohen’s Kappa values of 0.281 and 0.275, respectively.

Information from data-rich CYP450 isoforms helped the model learn representations for poorly represented isoforms. External evaluation using known inhibitors among 1,808 approved drugs produced recalls of 0.27 for CYP2B6 and 0.60 for CYP2C8, indicating moderate generalisation for later but limited performance for CYP2B6. Overall, the study demonstrates that multitask learning and missing-label imputation did not improve CYP450 inhibition prediction, especially when experimental data are limited. The relatively low external recall for CYP2B6 highlights the limitations of applying the approach to chemically diverse, data-poor CYP450 isoforms.

The Multimodal Encoding network (MEN) (Atwereboannah et al., 2025) framework further advanced multimodal prediction by combining molecular fingerprints, molecular graphs, and CYP450 protein sequences. The study used PubChem datasets covering CYP1A2, CYP2C9, CYP2C19, CYP2D6, and CYP3A4, with 51,546 compounds used for training/validation and 72,614 records reserved for testing. The proposed MEN architecture combined a Fingerprint Encoder Network (FEN), Graph Encoder Network (GEN), and Protein Encoder Network (PEN), followed by feature fusion, a Position Awareness Module, and a Vision Transformer with Dynamic Attention (ViTDA). The combined model achieved an average accuracy of 93.7% and a reported AUC of 98.5% compared to individual FEN, GEN, and PEN encoders. The model also generated ligand-level attention heatmaps that were compared with molecular docking interactions. Overall, the study demonstrates the potential of multimodal ligand–protein representations and attention mechanisms for CYP450 inhibitor prediction, although the complex architecture, limited protein-active site representation restricted to 42 binding residues, and relatively modest performance on the independent drug set remain important limitations.

## 8 Summary

CYP450 enzymes play a central role in the metabolism of xenobiotics and endogenous compounds and consequently influence the pharmacokinetics, efficacy, and safety of drugs. CYP450 inhibition has traditionally been regarded as a major liability because inhibition of drug-metabolising CYP450 enzymes can reduce metabolic clearance, increase systemic drug exposure, and lead to clinically significant drug-drug interactions (DDIs). However, CYP450 inhibition can also be therapeutically beneficial. Therefore, CYP450 inhibition should not be considered inherently detrimental, as its ultimate consequence depends on the isoform inhibited and the pharmacological and metabolic context.

In this review, we provided a comprehensive classification of CYP450 inhibitors by integrating their mechanisms of inhibition with the characteristic active site binding spectral patterns. This novel combined classification brings together concepts that are often discussed separately, is often difficult to understand for new readers and may be valuable for experienced researchers and practitioners of the field. It retains the base classification of CYP450 inhibitors into 1) Reversible and 2) Mechanism-based, that is evaluated using parameters such as *K*_I_, *k*_inact_, IC_50_, *K*_i_, *K*_m_, and V_max_. A thorough literature and database (ChEMBL) search revealed surprisingly that only four drugs are true reversible (non-substrate) inhibitors of CYP450. This observation suggests that true CYP450 reversible inhibition without metabolism may be rare among drugs and highlights the complexity of most of the inhibitors are also substrates. A modified version of the Cheng-Prusoff equation is used to explain the behaviour of substrates that act as inhibitors in alternate conformations in the active site. This behaviour is captured by the parameter *β* = *K*_m_/*K*_i,_ which is explained using appropriate CYP450 inhibitor drug examples from the literature. Specific examples mentioned in this review are binimetinib, which is predominantly a high residence-time reversible inhibitor followed by time-dependent metabolic activation and inactivation; mibefradil, which has a more balanced reversible inhibition.

Mechanism-based inhibitors (MBIs), classified into quasi-irreversible and irreversible types, exhibit inhibition either via direct coordination of heterocyclic nitrogen with heme-Fe or covalent modification of the heme or protein residues by reactive metabolites (RMs). This process is represented by a two-step mechanism involving reversible binding followed by irreversible coordination or covalent inactivation. The corresponding kinetic parameters, *K*_I_, *K*_i_, and *k*_inact_, are used to characterise inhibitor potency and inactivation efficiency, with the partitioning ratio (*K*_I_/K_i_) providing an assessment of the relative contributions of reversible and time-dependent inhibition. The combined consideration of binding affinity and inactivation rate therefore provides a more informative assessment of time-dependent inhibition than either parameter alone. A modified relationship *k*_inact_/*k*_off_ = (*K*_I_ */K*_i_ - 1) for estimating relative contributions of reversible/time-dependent binding thereby provides a practical alternative to conventional *k*_on_ and *k*_off_ measurements. This relationship may therefore provide a useful practical approach for interpreting inhibitor mechanism when direct determination of association and dissociation rate constants is not readily accessible. These observations (see examples discussed in the main text) indicate that binding affinity alone is insufficient to distinguish the behaviour of mechanism-based inhibitors, and that the relationship between affinity, metabolic activation, and inactivation rate must be considered together. Analysis of these kinetic parameters suggests that inactivation rate is primarily governed by the reactivity and mechanism of reaction for the reactive group, whereas binding affinity depends on the overall molecular structure.

CYP450 and their inhibitors are also characterised by the spectral features induced by various types of interactions and are classified as Type I or II inhibitors. The Type I inhibitors produce a characteristic difference spectrum (peak at 385–390 nm and trough at 418–425 nm). This spectral shift depends on the ligand’s ability to displace the coordinated water molecule. Despite their structural diversity, Type I ligands commonly possess hydrophobic aromatic or aliphatic frameworks, moderate-to-high lipophilicity, conformational flexibility, and a lack of strongly heme-coordinating heteroatoms typical of Type II inhibitors. Type II inhibitors stabilize low-spin state and produce a characteristic red shift in the Soret bands (absorbances at 425–435 nm and 390–410 nm). This spectral signature is therefore widely used as an indicator of direct heme-iron coordination and Type II inhibition.

Since these different classifications along with the FDA guidelines can be confusing, a modified workflow is proposed that recognizes this different inhibitory mechanism and can be useful for predicting DDI induced by CYP450 substrates, inhibitors, and inducers. The workflow incorporates reaction phenotyping, recombinant CYP450 and hepatocyte studies, determination of the fraction metabolised (*f*_m_), assessment of reversible and time-dependent inhibition, kinetic characterisation, and subsequent PBPK modelling. This modified workflow (Figures 10, 11) would require 4 steps less than the classical approach and can make the evaluation of DDI potential faster.

However, it is important to note that the mechanistic classification does not necessarily give insights into the effect of CYP450 inhibitor on clinical response, i.e., either beneficial (Friend) or detrimental (Foe). Therefore, potential parameters that can differentiate the CYP450 inhibitors as either Friend or Foe were analysed. Isoform selectivity and the effect of metabolism on drug action emerged as two important factors useful for differentiating CYP450 inhibitors. In our view, these factors are particularly important because the same inhibitory activity can produce either a favourable or unfavourable outcomes depending on which CYP450 isoform is inhibited and whether CYP450-mediated metabolism contributes positively or negatively to drug action. This analysis further indicates that isoform selectivity, including the ΔpIC50 criterion can provide a useful indication if the CYP450 inhibition is likely to be favourable (Friend) or detrimental (Foe). Additionally, other factors like systemic exposure, time-dependent inhibition, active metabolites, clearance, and other pharmacokinetic factors can modulate the response to CYP450 inhibitor administration. Consequently, a mechanistic label such as reversible, irreversible, or Type I or II is unlikely to be useful determining whether an inhibitor is a therapeutic Friend or Foe. Finally, computational prediction models for CYP450 inhibition published over the past 5 years were reviewed to assess the applications of ML, deep learning, LLM for prediction of CYP450 inhibitors. Although considerable progress has been made in predicting inhibition of individual CYP450 isoforms, direct prediction of CYP450 isoform selectivity remains unexplored. This represents an important limitation when computational predictions are intended to inform the therapeutic consequences of CYP450 inhibition. Overall, this review provides a clear and integrated understanding of CYP450 inhibitors by combining experimental protocols and computational prediction, which may be helpful in drug development.
